# H_2_S-responsive CuSe nanoparticles convert into CuSSe in situ with band gap modulation for oxygen self-supply and enhanced colon cancer phototherapy

**DOI:** 10.1186/s12951-025-03977-9

**Published:** 2025-12-31

**Authors:** Manoj Kandel, Arjun Sabu, Kai-Min Chan, Ramalingam Sharmila, Lakshminarayan Ramesan, Ming-Yin Shen, Yu-Fen Huang, Hsin-Cheng Chiu

**Affiliations:** 1 Department of Biomedical Engineering and Environmental Sciences, National Tsing Hua University, Hsinchu City, 300 Taiwan; 2 Institute of Analytical and Environmental Sciences, National Tsing Hua University, Hsinchu City, 300 Taiwan; 3 Department of Surgery, China Medical University Hsinchu Hospital, Hsinchu County, Zhubei City, 302 Taiwan

**Keywords:** Colorectal cancer, Copper selenide nanoparticles, In situ band gap modulation, Photocatalytic therapy, Hydrogen sulfide

## Abstract

Colorectal cancer (CRC) remains a significant challenge due to its aggressive nature and propensity for metastasis. This study presents a novel approach to CRC treatment by improved photothermal/photocatalytic/chemodynamic combination therapy in conjunction with modulation of the cancer microenvironment by scavenging endogenous hydrogen sulfide (H_2_S), a gasotransmitter overproduced in CRC cells. Copper selenide nanoparticles (CuSe NPs) scavenge H_2_S by sulfidation into copper sulfur selenium (CuSSe) NPs, leading to the in situ band gap modulation from 0.81 eV to 1.03 eV and formation of copper vacancies. These vacancies promote photothermal performances, while the change of band gap enables oxygen production via the oxidation of hydrogen peroxide at the valence band and superoxide radicals (O_2_^•−^) generation at the conduction band. Profound cytotoxic effect attained by the treatment of murine CT26 colon cancer cells with CuSe NPs and NIR-II laser irradiation corroborates the efficacy of the combined therapy alongside H_2_S depletion and disturbance of oxidative stress balance. Selective H_2_S depletion and formation of CuSSe NPs in H_2_S-rich colon cancer microenvironment efficiently promote the efficacy of the multimodality treatment and eradicate colon cancer in subcutaneous tumor-bearing mice. This strategy highlights the potential of in situ self-engineering nanotherapeutics with tumor microenvironmental remodeling for CRC therapy.

**Graphical Abstract:**

**Figure:**
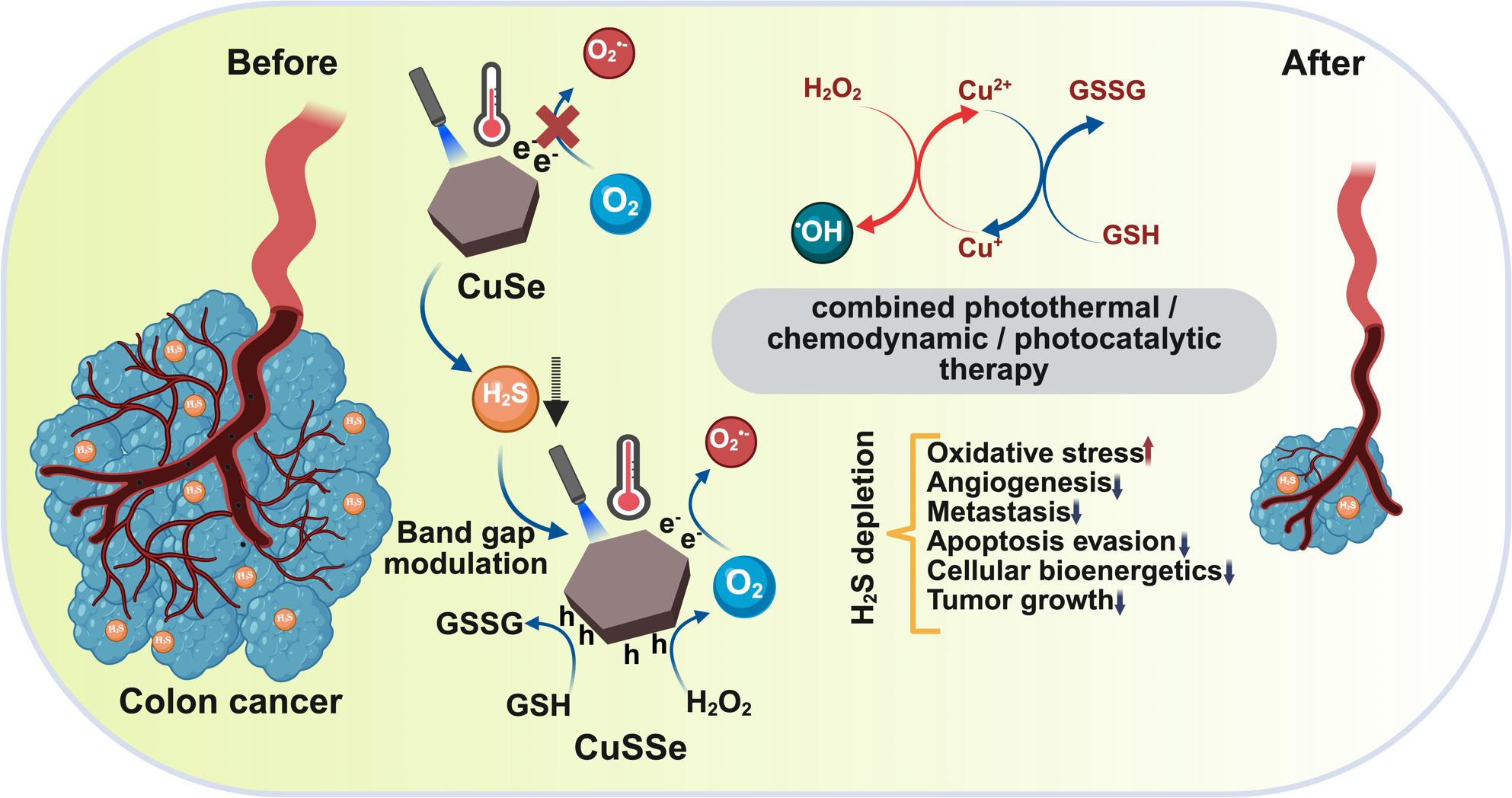
CuSe nanoparticles are developed as an in vivo self-transforming nanotherapeutics for cancer therapy. Upon reacting with overexpressed H2S in colon cancer, these nanoparticles are converted into CuSSe with modulated band gap and copper vacancies. The transformation enhances photothermal performance, oxygen production, and photocatalytic ROS generation with H2S depletion that remodels tumor microenvironment.

**Supplementary Information:**

The online version contains supplementary material available at 10.1186/s12951-025-03977-9.

## Introduction

Accounting for the third highest incidence among cancers and the second most frequent cause of cancer-related deaths, colorectal cancer (CRC) is known for its aggressive progression and strong metastatic capability [1]. A range of treatment options have been developed to prevent tumor relapse and distant metastasis in colon cancer patients. These strategies predominantly consist of curative resection of the primary tumor, frequently supplemented by chemotherapy and/or radiotherapy in advanced cancer stages [2]. Despite the advances in current treatment regimens, colon cancer therapy still faces challenges, often resulting in suboptimal clinical outcomes due to debilitating side effects. To address the shortcomings of existing treatment modalities, multifaceted approaches incorporating novel therapeutic strategies are in great demand [3].

It has been realized that intracellular hydrogen sulfide (H_2_S), produced primarily by cystathionine-β-synthase (CBS), is an important gasotransmitter that is closely linked to several physiological and pathological processes [4]. At physiological concentrations, H_2_S significantly influences cytoprotective pathways due to its ability to reduce inflammation, neutralize oxidative stress, and inhibit apoptosis [5]. By contrast, it has been discovered that aberrant H_2_S generation in biological systems is associated with the development of numerous illnesses, including cancers, Alzheimer’s disease, liver cirrhosis, and other inflammation-related diseases [6]. Among all cancers, CRC is known to have a high level of H_2_S (0.01-3 mM) due to overexpression of the CBS enzyme [7]. Aberrant H_2_S production contributes to the onset of CRC by activating cellular bioenergetics and the progression by enhancing angiogenesis, tumor proliferation, and metastasis [8]. Developing H_2_S-responsive therapeutic nanoagents can therefore be an effective strategy for achieving colon tumor-specific therapy. However, the strategic scavenging of endogenous H_2_S has not yet been extensively investigated. The overexpressed endogenous H_2_S was used for the conversion of Cu_2_O to Cu_9_S_8_ to achieve strong photoacoustic (PA) imaging and increased photothermal property [9]. A similar idea was adopted in a study where H_2_S-triggered Ag@Cu_2_O was used to diagnose and treat CRC. Ag@Cu_2_O, in the presence of H_2_S, converts to Ag@Cu_9_S_8,_ resulting in improved Near-infrared (NIR) absorbance, PA imaging, and photothermal property [10]. These studies focused on using H_2_S as a turn-on theragnostic agent and highlighted the potential of tumor microenvironment (TME) modulation in colon cancer.

Transition metals (Cu, Mn, and Fe) and chalcogenide elements (S, Se, and Te) have been widely studied for their roles in electrochemistry, catalysis, energy storage, and biomedical applications due to their excellent thermal conductivity, catalytic efficiency, and unique optical properties [11, 12]. In addition, transition metal chalcogenides have often been adopted in ion exchange reactions and transformation into ternary compounds, which offer a broad spectrum for tailoring the inherent optical and electronic properties. These ternary nanoparticles (NPs) possess unique properties and characteristics that are inherent to their parent binary compounds [13]. Substituting or doping with foreign ions to induce modification of the electronic configuration of a material is particularly effective in tailoring the bandgap of semiconductors, which is a crucial parameter that determines their optical and electrical properties [14–17]. In general, lattice contraction occurs when larger ions substitute for smaller ions and vice versa [18]. Compression of the unit cells was observed when sulfur replaced selenium in Cu_3_Se_2_ NPs [17]. In addition, the band gap (Eg) of Cu_3_Se_2_ is gradually increased due to increased structural distortion caused by a higher concentration of doped sulfur ions substituting selenium within the crystal lattice. Consequently, doping-induced enlarged band gap for improved photocatalytic reaction by preventing electron-hole recombination is highly promising in the field of combination cancer therapy. Furthermore, copper selenide (CuSe) NPs featuring the Cu composition hold the potential to be an ideal candidate to generate reactive oxygen species (ROS) via Fenton-like reaction and reduce glutathione (GSH) to disturb complex redox homeostasis. Selenium is an essential trace element with important biological functions. Selenium-containing NPs have been found to have selective anticancer propensity while sparing healthy cells [19]. CuSe NPs thus stand out as an excellent choice for achieving effective curative effects in cancer nanomedicine due to their ability to support multimodal treatment strategies. While it has been reported as a photothermal agent, its potential for enhanced ROS generation via band gap modulation in vivo has still not been explored [20]. In an attempt to develop drug-free NPs for cancer treatment, a direct Z-scheme heterojunction structure based on FeS_2_ core and Fe_2_O_3_ shell was synthesized, where NIR generated electrons reduced O_2_ to O_2_^•−^ at the valence band (VB) and holes oxidized H_2_O to •OH at the conduction band (CB) [21]. It led to the production of enormous ROS and remodeling of the tumor microenvironment for facilitated cancer cell apoptosis. While increasing attention has been paid to the heterojunction NPs for their potential in multimodality cancer treatments, the synthesis and uniformity of heterojunctions are challenging. Therefore, designing NPs that enhance photocatalytic properties within the tumor microenvironment (TME) while modulating or reversing its pro-cancer characteristics remains challenging, yet urgently needed.

Considering these factors, hexagonal disc-like CuSe NPs were prepared and characterized with high reactivity toward the overexpressed H_2_S in colon cancer. While endogenous H_2_S was depleted with the reaction, sulfur (S) was doped within CuSe NPs, giving rise to new copper sulfur selenide (CuSSe) NPs with copper vacancies and a modulated band gap. The resulting CuSSe NPs showed superior photothermal and photocatalytic performances compared to CuSe NPs. Albumin was used for surface functionalization of CuSe NPs to improve colloidal stability, facilitate transport to the tumor region, and increase cellular uptake by SPARC overexpressing cancer cells [22]. The presence of both copper ions (Cu^+^ and Cu^2+^) in the NP structure improves catalytic properties and thus increases ROS generation. More intriguingly, S doping induces copper vacancies and simultaneously modulates the band structure in CuSSe NPs. The shifting of the CB potential near the reduction potential of O_2_ enables its reduction into O_2_^•−^. At the same time, the VB potential exceeds the oxidation potential of H_2_O_2_, catalyzing the production of O_2_, thus establishing CuSSe NPs as an oxygen self-supplying nanoplatform for enhanced ROS generation. The administration of CuSe NPs by intravenous (IV) injection exhibits enhanced accumulation in the tumor of mice bearing CT26 carcinoma, effective H_2_S depletion in tumor, improved photothermal property, and high capability of undergoing photocatalyzed ROS generation reactions (Scheme 1). This study highlights the potential of in situ self-engineering nanoplatform for tumor microenvironment remodeling and of combined photothermal therapy (PTT), chemodynamic therapy (CDT), and photocatalytic therapy (PCT) for cancer treatment.

**Fig. 1:**
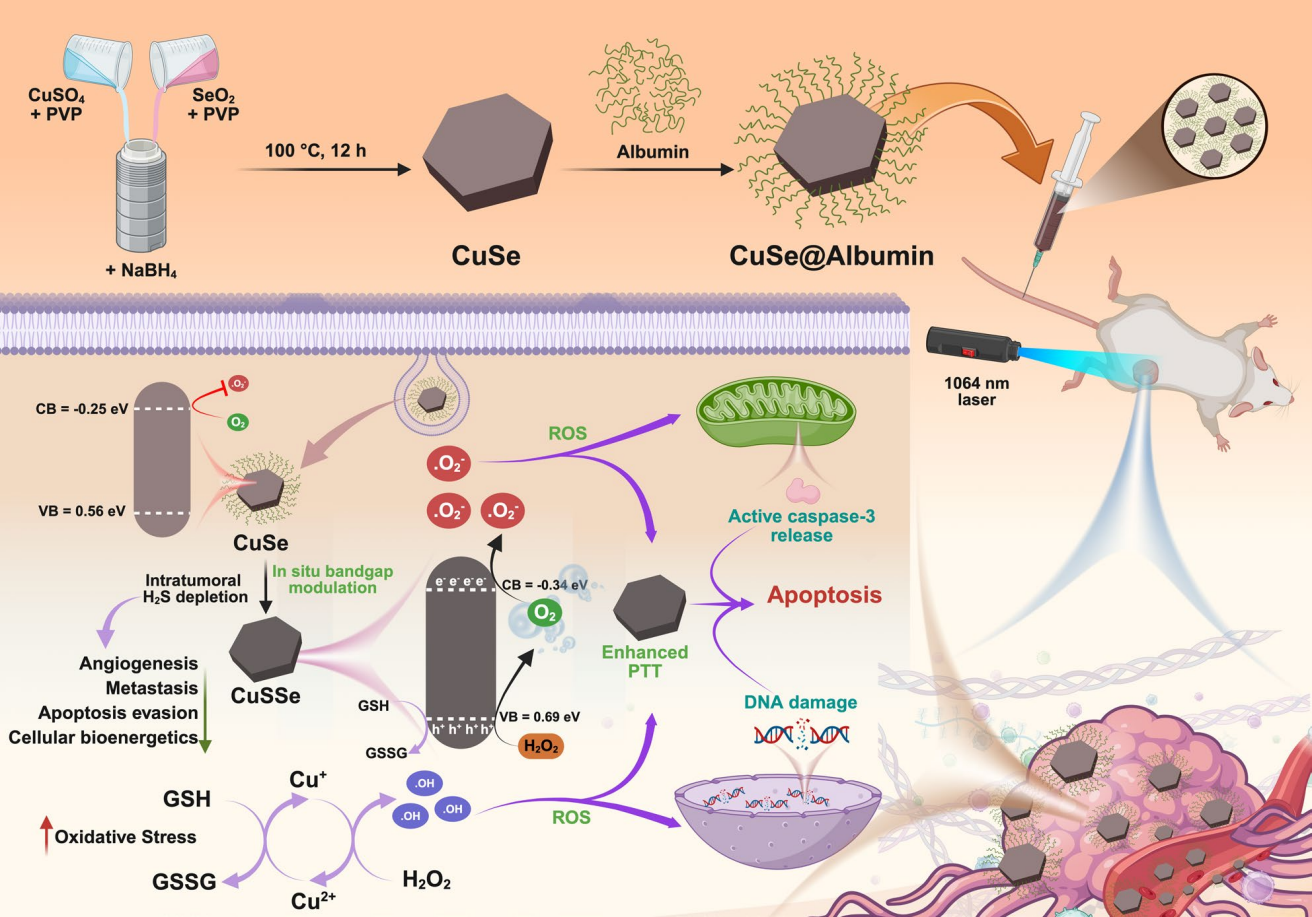
Schematic illustration of the synthesis and the therapeutic mechanisms of CuSe NPs operated by reaction with H_2_S and conversion into CuSSe NPs in situ, enabling H_2_S depletion and enhanced photothermal, chemodynamic, and photocatalytic effects for synergistic cancer therapy

## Results and discussion

### Preparation and characterization of CuSe NPs

**Fig. 2:**
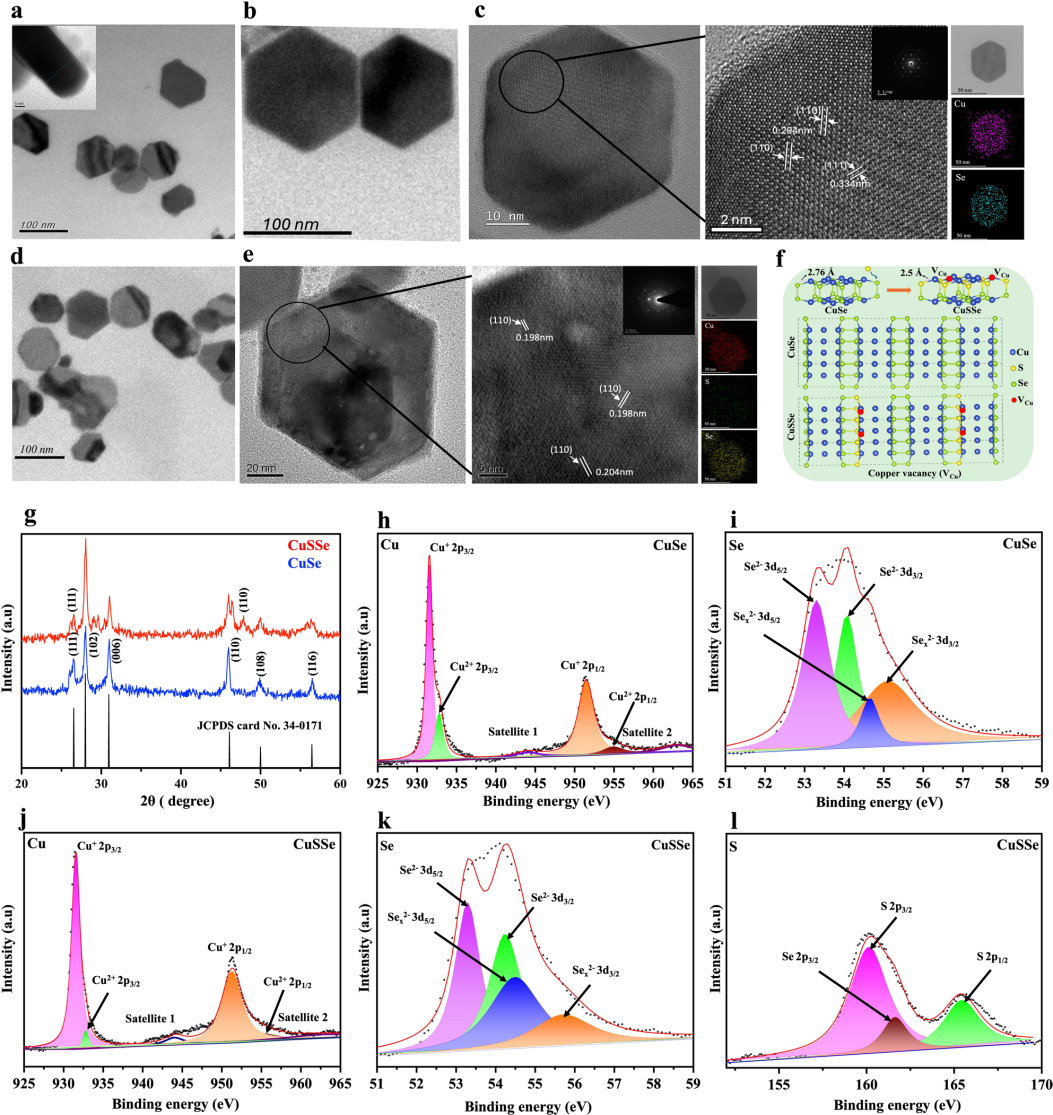
TEM images of (**a**) CuSe NPs (inset as a side view of the NPs) and (**b**) CuSe@albumin NPs. (**c**) HRTEM image of CuSe NPs (SAED pattern and EDX elemental mapping of Cu and S in CuSe NPs included). (**d**) TEM image of CuSe NPs after reacting with 1 mM H_2_S. (**e**) HRTEM image of CuSSe NPs (SAED pattern and EDX elemental mapping of Cu, S and Se in CuSSe NPs included). (**f**) Schematic crystal structures of CuSe and CuSSe NPs. (**g**) XRD pattern of CuSe NPs and CuSSe NPs. Deconvoluted XPS spectra of (**h**) Cu 2p and (**i**) Se 3d of CuSe NPs and (**j**) Cu 2p, (**k**) Se 3d, and (**l**) S 2p of CuSSe NPs

CuSe NPs were synthesized via a facile hydrothermal method for the combined PTT/CDT/PCT against colon cancer. CuSO_4_ and SeO_2_ at an equimolar ratio were reduced separately by NaBH_4_ at room temperature. The prepared solutions were mixed thoroughly, and the reaction was conducted in a sealed autoclave at 100 °C for 12 h. Polyvinyl alcohol (PVA) was used in the reaction as a surfactant and shape-directing agent. Morphological assessment by transmission electron microscope (TEM) revealed the hexagonal disc-shaped structure of CuSe NPs with a mean diameter of 52 ± 8.26 nm and a thickness of 12 ± 2 nm (Fig. 2a). To improve aqueous dispersion and cellular uptake of CuSe NPs, albumin was coated onto the surfaces via amine-metal ion interaction. The hexagonal disc morphology of CuSe NPs remained unchanged after the protein coating (Fig. 2b). Dynamic light scattering (DLS) measurements demonstrated a reduction in polydispersity index from 0.202 to 0.108 with a mean hydrodynamic diameter of approximately 128 nm (Fig. S1a in Supplementary Information (SI)). Zeta potential analysis showed a shift from − 13 ± 3.4 mV to -19 ± 2.7 mV following the protein coating (Fig. S1b). Albumin coating significantly improved the dispersion of CuSe NPs in PBS and cell culture media (RPMI) over 48 h (Fig. S1c). These findings suggest improved colloidal stability through electrostatic shielding, thereby reducing aggregation and improving dispersion. High-resolution TEM (HRTEM) image illustrated in Fig. 2c shows distinct lattice fringes with an interplanar spacing of 0.33 nm and 0.20 nm, corresponding to the (111) and (110) crystallographic planes of hexagonal CuSe NPs, respectively (JCPDS card No. 34–0171) [23]. Additionally, elemental mapping confirmed the even distribution of Cu and Se in the CuSe NPs, further validating the successful synthesis of the NPs (Fig. 2c).

The sulfidation of CuSe NPs was examined by their conversion to CuSSe NPs after exposure to H_2_S. TEM analysis revealed no significant changes in the morphology or size of NPs after H_2_S treatment (Fig. 2d). However, HRTEM analysis highlighted the appearance of a new lattice fringe with an interplanar spacing of 0.19 nm, which can be assigned to the (110) planes of CuS, in addition to the original characteristic fringes of hexagonal CuSe (Fig. 2e). The presence of the additional CuS-related lattice fringe and the observed lattice compression confirm successful S doping into the CuSe lattice. Substitution of Se atoms by S atoms occurs due to their comparable atomic radii and similar chalcogen chemistry, with S atoms occupying Se lattice sites without disrupting the overall hexagonal framework [24, 25]. The incorporation of slightly smaller S atoms, nevertheless, induces local lattice contraction, creating strain within the crystal structure. To accommodate this strain and maintain lattice stability, Cu vacancies are formed, thereby relieving lattice distortion and preserving charge neutrality. Figure 2f illustrates this structural adaptation with a schematic crystal model to highlight the S substitution and the resulting Cu vacancy (V_Cu_) formation within the doped CuSe lattice. Sulfur atoms substitute selenium atoms in the CuSe lattice, forming Cu–S bonds with an average bond length of approximately 2.5 Å, which is shorter than the original Cu–Se bond length (2.76 Å). This difference in bond lengths induces lattice distortion and local strain, influencing the band structure and defect formation energetics [26–28]. X-ray diffraction (XRD) analysis showed a new peak at 47.

81°, a prominent peak for the (110) plane of CuS (JCPDS No. 06464) (Fig. 2g). As sulfur atoms were doped into the copper selenide lattice, there were shifts in the 2θ values of the (102) and (200) planes observed from 27.98° and 30.97° to 28.23° and 31.02°, respectively. These changes toward higher 2θ indicate compression of the crystal unit cell, while confirming the successful substitution of selenium by sulfur atoms. Elemental mapping and energy-dispersive X-ray spectroscopy (EDX) analysis as shown in Fig. 2e and S1d, respectively, confirm the presence of S along with Cu and Se, indicating successful S doping (7%) and the formation of CuSSe NPs after reacting with H_2_S. This transformation from CuSe to CuSSe NPs upon exposure to H_2_S at room temperature highlights the potential for these NPs to serve as an H_2_S scavenger in the TME of colon cancer.

X-ray photoelectron spectroscopy (XPS) measurement was used to analyze the surface elemental composition and chemical state of CuSe NPs. The line spectra of XPS revealed characteristic signals corresponding to Cu and Se, confirming their presence in CuSe NPs (Fig. S1e). Deconvolution of the Cu 2p spectrum identifies two distinct peaks at 931.5 eV, and 951.3 eV, ascribed to Cu 2p3/2 and Cu 2p1/2, respectively (Fig. 2h). A split orbit calculated at 19.6 eV confirms the presence of Cu^1+^ within the sample [17]. The peaks observed at 932.9 eV and 955.0 eV correspond to Cu 2p3/2 and Cu 2p1/2, respectively, thereby confirming the presence of Cu in the + 2 oxidation state, with the characteristic satellite peaks of Cu^2+^ appearing at 944.0 eV and 962.9 eV [29]. Atomic percentage analysis revealed that Cu^1+^ and Cu^2+^ accounted for approximately 83% and 17% of the atomic composition within the CuSe NPs, respectively. Figure 2i shows the high-resolution spectrum of Se 3d, exhibiting two well-defined doublets. The first observed at binding energies of 53.29/54.07 eV, corresponds to the Se 3d5/2 and Se 3d3/2 states of Se^2−^ in selenides [30]. The second doublet, located at 54.65/55.11 eV, likely arises from Se^2−^ existing in a different chemical context or diselenide [31]. The Cu 2p3/2 peak remains fixed at 931.52 eV (Fig. S2a), demonstrating that the Cu^+^ oxidation state and its coordination environment is not changed during sulfidation, and that S substitution occurs primarily within the anion (Se/S) sublattice rather than at Cu sites. Meanwhile, the Se 3d3/2 peak shifts from 54.07 eV in CuSe to 54.29 eV in CuSSe NPs. This shift suggests electron deficiency at the Se sites, which is likely due to the incorporation of more electronegative S atoms into the lattice. The higher binding energy Se components at 54.65 and 55.11 eV, often attributed to selenium oxides or diselenides, show noticeable shifts, confirming that these states are particularly sensitive to the anion-sublattice modification introduced by S doping (Fig. S2b). In the S 2p XPS spectrum, two distinct peaks were observed at binding energies of 162.5 eV and 164.3 eV, corresponding to the S 2p3/2 and S 2p1/2 components, respectively (Fig. 2l). These peaks confirm the presence of sulfide ions within the sample. Additionally, a binding energy at 159.4 eV was detected for the Se 2p3/2 signal (Fig. 2k), which could be linked to the selenide oxide state. Signal overlap between sulfur and selenium near 159 eV implies that both elements contribute to the observed peaks, as the S 2s peak partially obscures the Se 2s peak [32].

### Band gap modulation and photocatalytic ROS generation

Incorporating sulfur as a dopant into CuSe NPs inevitably introduces a significant increase in free electron concentration, resulting in a marked alteration in the electronic properties of the crystalline structure. It is postulated that this increase in electron density raises the Fermi level close to or above the CB minimum, resulting in the energy states at the low edge of the CB being occupied. As a consequence, the interband optical transitions from the VB are restricted only to higher-energy states within the CB, which effectively increases the optical bandgap. This apparent bandgap widening has been referred to as the Burstein-Moss effect [33, 34]. In addition, it is also noteworthy that sulfur doping in CuSe NPs promotes the formation of copper vacancies within the lattice. These Cu vacancies function as acceptor states, increasing hole concentration and significantly improving both optical absorption and electrical conductivity. Sulfur doping in CuSe NPs not only increases the carrier concentration by introducing free electrons but also promotes copper vacancy formation, both of which contribute to enhanced electrical properties.

**Fig. 3:**
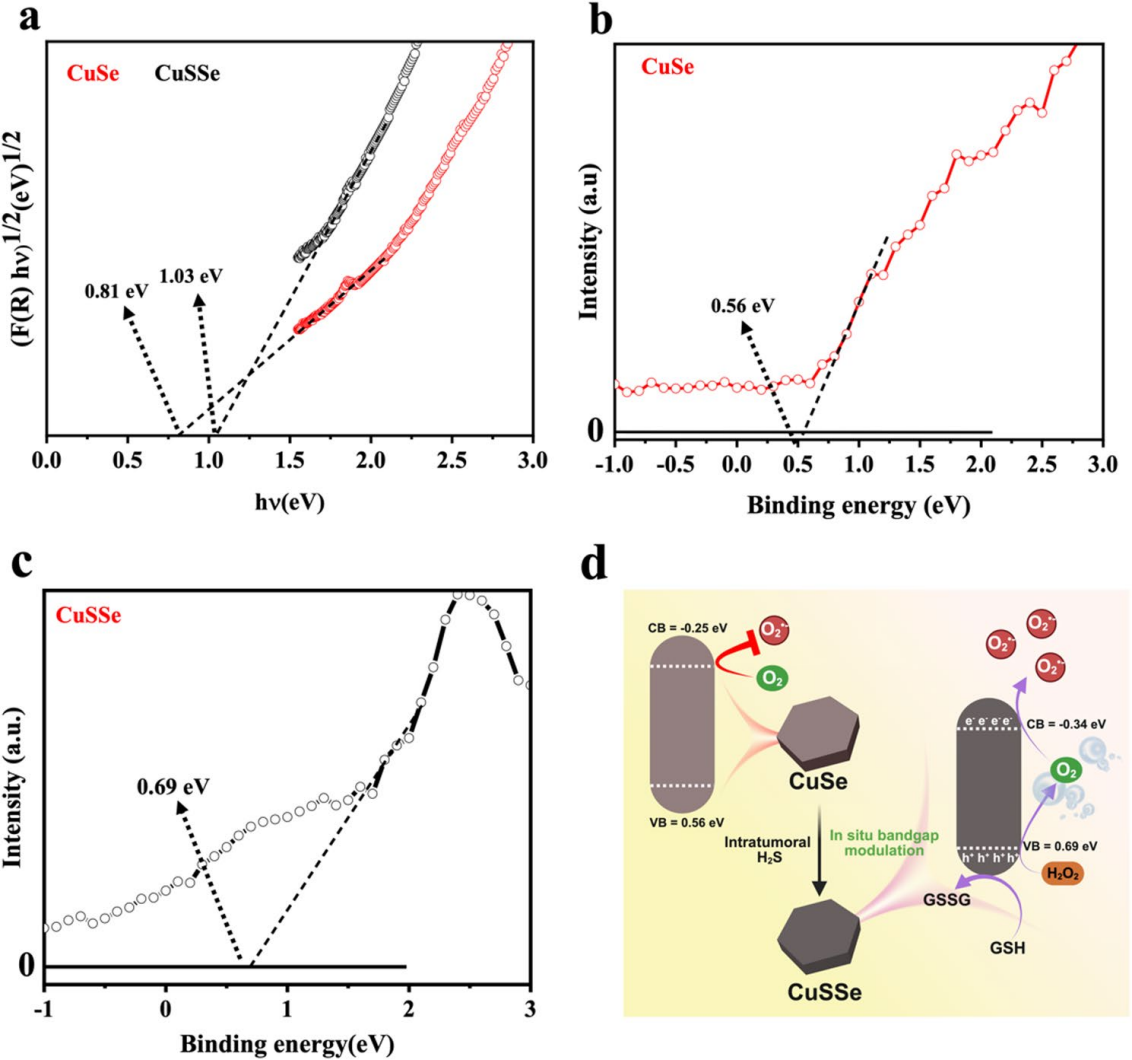
(**a**) Tauc’s plots for band gap energies of CuSe and CuSSe NPs calculated from DRS. Valence band potentials of (**b**) CuSe and (**c**) CuSSe NPs calculated from XPS. (**d**) Schematic illustration of photocatalytic oxygen reduction by NIR irradiation of CuSSe NPs after band gap modulation via S doping into CuSe NPs

Zafar et al. have reported an increase in the band gap of Cu_3_Se_2_ NPs with S doping due to electron-amplified lattice deformation in the NPs. The band gap widening induced by S doping effectively reduces the electron–hole recombination rate, thereby leading to enhanced photoelectrochemical activity [17]. CuSe NPs synthesized in this work were found capable of reacting with H_2_S, transforming themselves into CuSSe NPs as aforementioned. The optical band gaps and band edge potentials of CuSe and CuSSe NPs were evaluated with diffuse reflectance spectroscopy (DRS) measurement and XPS valence band estimation referenced to the Fermi level (0 eV), respectively, while the CB was derived based on the calculated band gap values. The E_g_ of CuSe NPs, estimated by Tauc plots derived from the DRS measurements, was found to be 0.81 eV, whereas, after sulfur doping, the Eg of CuSSe NPs was increased to 1.03 eV (Fig. 3a). This widening of the band gap is consistent with previous studies [17, 25], confirming the successful incorporation of S atoms through the reaction with H_2_S. From the XPS VB estimation, the VB potential of CuSe NPs was determined to be 0.56 eV (Fig. 3b). With S atoms doped, the VB potential shifted to 0.69 eV (Fig. 3c). Given that the CB potential was calculated as E_CB_​ = E_VB_​ - E_g_, the CB potential of CuSe NPs was estimated to be -0.25 eV which further shifted to -0.34 eV for CuSSe NPs after sulfidation. Such a shift in CB potential to -0.34 eV renders it closer to the reduction potential of O_2_ to O_2_^•−^ radicals (O_2_ ◊O_2_^•−^, -0.33 eV) [35]. These findings suggest that the band structure of CuSe NPs can be precisely tuned in an H_2_S-rich tumor microenvironment, offering potential applications in selective PCT under NIR irradiation. Figure 3d schematically illustrates the H_2_S-triggered transformation of CuSe into CuSSe and its implications for PCT. The conversion of CuSe into CuSSe via sulfur doping in the presence of H_2_S results in band gap widening via the Burstein-Moss effect. Notably, the shift of CB potential from − 0.25 eV to -0.34 eV enables CuSSe, but not CuSe, to generate O_2_^•−^ under 1064 nm NIR irradiation. This H_2_S-responsive transformation facilitates selective ROS generation in an H_2_S-rich tumor microenvironment, offering a precise therapeutic strategy.

### In situ response evaluation of the CuSe NPs

**Fig. 4:**
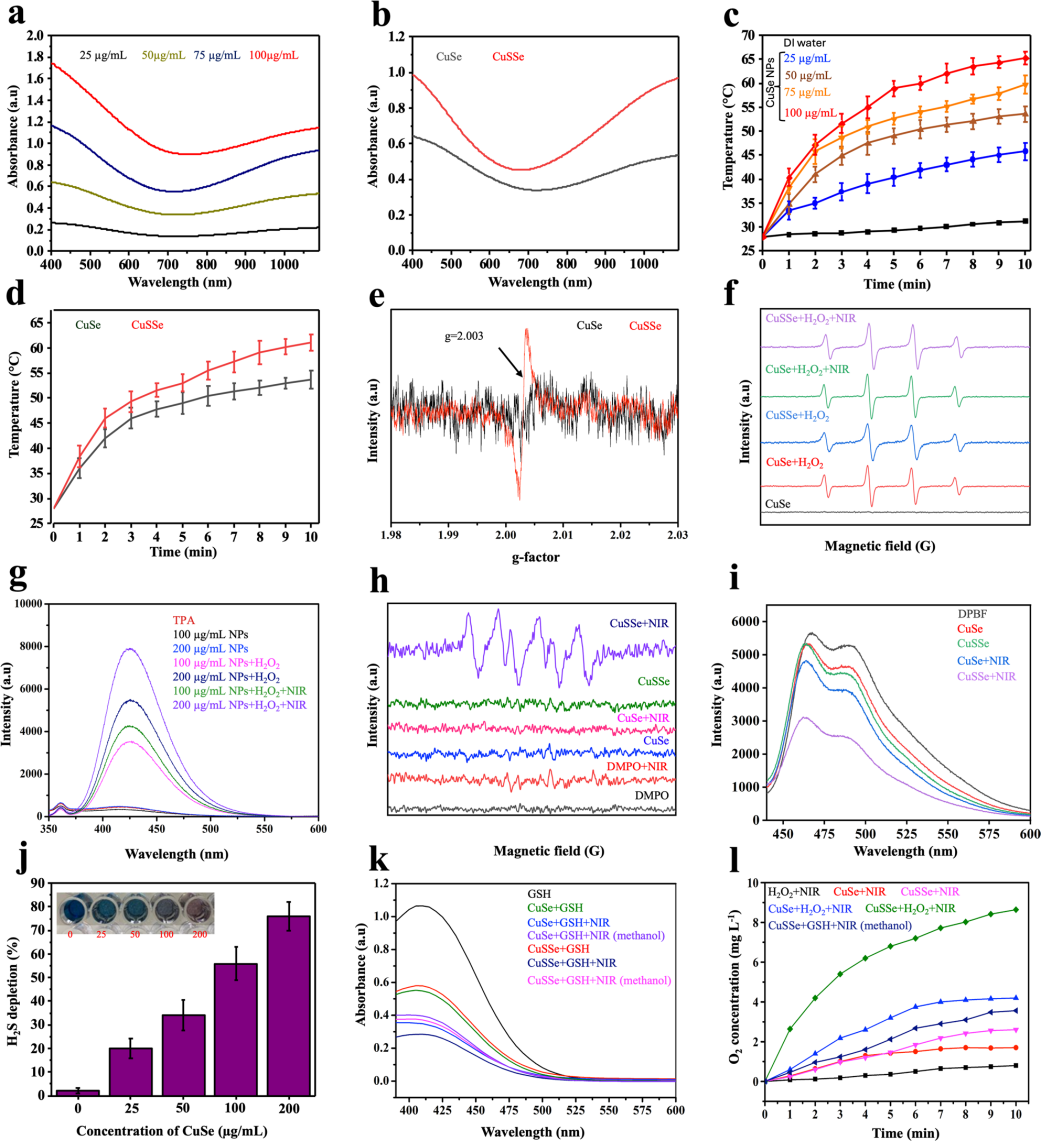
(**a**) UV-Vis absorption spectra of CuSe NPs of different concentrations. (**b**) UV-Vis absorption spectra of CuSe NPs (50.0 µg/mL) before and after reaction with H_2_S (1.0 mM). (**c**) The temperature profiles of aqueous dispersions of CuSe NPs with different concentrations subjected to NIR irradiation for 10 min. (**d**) The temperature profiles of CuSe NPs (50 µg/mL) by NIR irradiation before and after reaction with H_2_S (1.0 mM). (**e**) EPR spectra showing copper vacancy in CuSSe NPs. (**f**) EPR spectra of •OH radicals produced by the reactions of CuSe and CuSSe NPs with H_2_O_2_, respectively. (**g**) Fluorescence spectra of TPA for detecting the production of •OH under different reaction conditions. (**h**) EPR spectra of O_2_^•−^ radicals produced by photocatalytic O_2_ reduction with NIR irradiation of CuSSe NPs. (**i**) Fluorescence spectra of DPBF under different reaction conditions. (**j**) H_2_S depletion by CuSe NPs of varying concentrations with the methylene blue assay. Inset photos of MB colors attained from different NP concentrations used. (**k**) UV–Vis spectra of TNB produced from the reduction of DTNB (Ellman’s reagent) by GSH under various reaction conditions. (**l**) O_2_ generation profiles under different reaction conditions. The concentration of H_2_O_2_ at 1.0 mM and NIR irradiation for 10 min at 1064 nm (1.0 W/cm^2^) were employed for the respective experiments

Theranostic agents featuring absorbance in the NIR-I (750–950 nm) or preferably NIR-II (1000–1700 nm) regions, collectively known as biological transparency windows, are of great importance for biomedical imaging and photo-triggered therapies due to minimal tissue absorption and enhanced penetration depth. Notably, the light within the NIR-I region permits tissue penetration in the range 1–6 mm and the NIR-II region extends this capability to depths of up to 20 mm, rendering the NIR-II window more favorable for deep tissue penetration and photoactivated therapies [36]. Figure 4a shows the absorbance spectra of CuSe NPs of varying concentrations, emphasizing the significant absorption in the NIR regions. The results validate the potential of CuSe NPs in photothermal therapy and imaging applications. It is well known that CuSe is a p-type semiconductor with a relatively high concentration of mobile holes, leading to strong free carrier absorption as characterized by its local surface plasmonic resonance (LSPR) behavior [37]. Following sulfidation by H_2_S, a slight blue shift and an overall increase in absorbance was observed in CuSSe NPs (Fig. 4b). As aforementioned, with sulfur replacing selenium in the CuSe lattice, it leads to local crystal distortions and dislocation of neighboring copper atoms in crystal lattice. Vacancies thus develop. These vacancies are responsible for increased light scattering frequency along with enhanced light absorption. Given the wide absorption range across the entire spectrum, including the NIR region, the photothermal properties of the CuSe NPs were assessed by irradiating aqueous dispersions of the NPs at different concentrations with an NIR-II laser at 1064 nm (1.0 W/cm^2^, 10 min). The temperature of aqueous NPs dispersion (100 µg /mL) increased by 38 °C within 10 min in comparison with only about 3 °C for DI water (Fig. 4c). Significant concentration and laser power density-dependent changes in temperature were observed (Fig. 4c and S3a), highlighting excellent NIR-II-mediated hyperthermia performance. The photothermal effect of CuSe NPs after being transformed into CuSSe NPs by sulfidation with H_2_S was also evaluated. CuSe NPs in aqua was subjected to the treatment with H_2_S (1.0 mM) for 3 h and laser irradiation at 1064 nm for 10 min. In contrast to the temperature rise of CuSe NPs (50 µg/mL) from 27 °C to 53 °C, the temperature of CuSSe NPs at the same concentration reached 61 °C (Fig. 4d). This is ascribed to improved LSPR property by additional vacancies created following S doping. The excellent photothermal stability of CuSe NPs was demonstrated by the essentially unchanged temperature profiles during repeated ON/OFF cycles of NIR irradiation with each ON and OFF duration of 10 min for five cycles (Fig. S3b). This vacancy-mediated structure modulation not only improved NIR-I and NIR-II absorption but also translated into a marked increase in photothermal conversion efficiency, rising from 65.33% for CuSe NPs to 71.49% for CuSSe NPs under identical irradiation conditions (Fig. S3b and c). The data signify the sound photothermal performance of both CuSe and CuSSe NPs by NIR-II irradiation for further uses as photothermal therapy against malignant neoplasms.

Electron paramagnetic resonance (EPR) spectroscopy further supports the presence of copper vacancies, as evidenced by the characteristic signal observed at g = 2.03, which is attributed to the unpaired electrons localized at Cu vacancy sites (Fig. 4e**).** The ROS generation from CuSe and CuSSe NPs in the presence of H_2_O_2_ was verified by EPR using 5,5-dimethyl-1-pyrroline n-oxide (DMPO) as the spin trap as shown in Fig. 4f. It is ascribed to the Fenton-like reaction involving the oxidation of Cu^+^ in NPs with H_2_O_2_ to generate •OH radicals. With the NIR irradiation at 1064 nm, an increase in the EPR peak intensity was observed, although no significant difference was detected between the CuSe and CuSSe NPs. This suggests that the •OH radicals are primarily produced from Fenton-like reactions. Following this terephthalic acid (TPA), a fluorescence probe was used to detect •OH radicals. Upon reaction with •OH, TPA undergoes hydroxylation, leading to the formation of 2-hydroxyterephthalic acid, which exhibits fluorescence at 425 nm. The concentration-dependent increase in the fluorescence intensity from TPA at 425 nm was observed which was further elevated in the presence of NIR irradiation (Fig. 4g). The increased ROS generation by NIR irradiation is ascribed to the accelerated Fenton-like reaction with increasing the reaction temperature and additional photocatalytic action induced by plasmonic holes in CuSe NPs [38]. EPR spectroscopy was also employed to detect the generation of O_2_^•−^ radicals, using DMPO as a spin-trapping agent in methanol. The EPR spectra revealed clear production of O_2_^•−^ radicals upon NIR irradiation of CuSSe NPs, whereas no O_2_^•−^ radicals were detected following the irradiation of CuSe NPs (Fig. 4h). 1,3-Diphenylisobenzofuran (DPBF) was used as a probe via photobleaching reaction with O_2_^•−^ radicals. A significant decrease in the fluorescence signal was observed in CuSSe + NIR group, confirming efficient O_2_^•−^ production under NIR irradiation (Fig. 4i). This signifies the band gap change and CB shift toward a more negative value by sulfidation of CuSe NPs with H_2_S and transformation to CuSSe NPs, capable of reducing O_2_ to O_2_^•−^ radicals.

It has been shown that CRC is often associated with elevated H_2_S levels, which promote cancer cell proliferation, tumor invasion/metastasis, and angiogenesis [6, 39]. H_2_S has been found capable of scavenging cytotoxic ROS, thereby impairing the efficacy of photo- and chemodynamic therapies [40]. Several approaches to reducing H_2_S levels in CRC as an auxiliary modality for cancer treatment have been reported [41–43]. The H_2_S depletion by CuSe NPs was examined with the methylene blue (MB) assay [44]. Na_2_S (1.0 mM) was used as an H_2_S donor. As illustrated in Fig. 3j, H_2_S was depleted significantly with a strong dependence on the NP concentration. CuSe NPs at a concentration of 200 µg/mL effectively depleted 76% of H_2_S within 30 min, highlighting their high reactivity toward H_2_S. Unlike traditional anion exchange processes requiring elevated temperatures or complex reaction conditions, the anion exchange reaction of CuSe with H_2_S occurring at room temperature and its transformation into CuSSe NPs represents a novel approach with promising therapeutic potential. This process allows the in situ synthesis of CuSSe NPs within tumor and enables the depletion of endogenous H_2_S often overexpressed in CRC. It is also noteworthy that the VB potentials of both CuSe and CuSSe NPs are higher than the oxidation potential of GSH (≥ 0.24 eV for GSH oxidation). As a result, GSH can be depleted not only through its reaction with released Cu^2+^ ions, but also via oxidation catalyzed by photoexcited holes in the VB. While being oxidized to GSSG, GSH acts as a natural sacrificial agent to consume photoexcited holes, which reduces charge recombination and consequently improves ROS production from the electrons in the CB. GSH depletion was quantified using Ellman’s reagent (DTNB) [45] with various NP treatments, including CuSe + GSH, CuSe + GSH + NIR, CuSe + GSH + NIR (methanol), CuSSe + GSH, CuSSe + GSH + NIR, and CuSSe + GSH + NIR (methanol), where the CuSe NP concentration of 75 µg/mL was used (Fig. 4k). Both CuSe and CuSSe exhibited moderate GSH depletion, primarily due to the redox reaction between GSH and Cu^2+^. Upon NIR irradiation, GSH depletion increased significantly in both cases, with CuSSe showing greater conversion efficiency, indicating the catalytic attribution of photoexcited holes to GSH oxidation. To further confirm the role of holes, methanol, a known hole scavenger was introduced. A significant decrease in GSH depletion was observed in the CuSSe + NIR treatment with methanol, while only a slight change was noted for CuSe + NIR under the identical conditions, suggesting that CuSSe NPs under NIR irradiation produce more active photoexcited holes with the increased catalytic power to GSH oxidation compared to the CuSe counterpart. The positive shift in the VB potential of CuSe NPs from 0.56 eV to 0.69 eV after sulfidation also signifies the improved ability of the electrons to catalyze O_2_ generation from H_2_O_2_, given that the oxidation potential for H_2_O_2_ to O_2_ is ≥ 0.68 eV [46]. As expected, significant O_2_ production was observed in the CuSSe + H_2_O_2_ + NIR group, indicating efficient hole-mediated oxidation of H_2_O_2_ (Fig. 4l). To validate the involvement of valence band holes, methanol was introduced as a hole scavenger, resulting in a significant reduction in O_2_ generation from CuSSe NPs. As a consequence, the band gap modulation induced by sulfidation of CuSe NPs enables the NPs to serve as an oxygen self-generating platform for improved photocatalytic ROS production.

### In vitro therapeutic efficacy of CuSe NPs

**Fig. 5:**
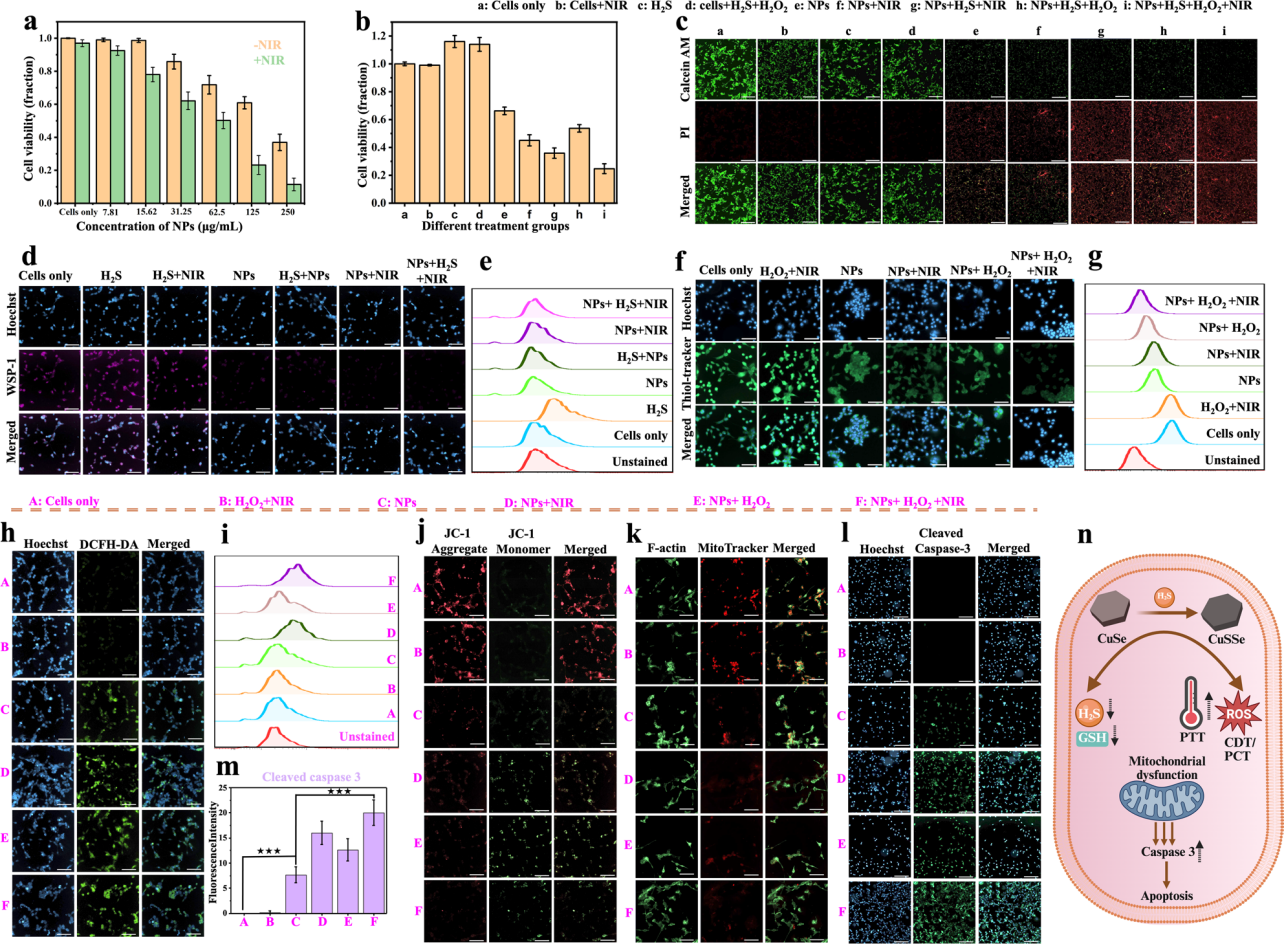
In vitro cytotoxicity of CuSe NPs against CT26 colon cancer cells. (**a**) Cell viability after the treatment with CuSe NPs of varying concentrations with and without NIR irradiation (1064 nm, 1.0 W/cm^2^ for 10 min). (**b**) Cell viability of CT 26 cancer cells receiving different treatments, including PTT (NPs + NIR), CDT (NPs + H_2_O_2_), PTT/CDT (NPs + H_2_O_2_ + NIR), PTT (enhanced)/PCT (NPs + H_2_S + NIR), and PTT (enhanced)/CDT/PCT (NPs + H_2_S + H_2_O_2_+NIR). (**c**) Live-dead assay after different treatments (scale bars 100 μm). Fluorescence images of CT26 cells with different treatments by staining with (**d**) WSP-1 (H_2_S staining). (**e**) Flow cytometric analysis of intracellular H_2_S levels in CT26 cells following different treatments. (**f**) ThiolTracker violet staining for GSH. (**g**) Flow cytometric analysis of intracellular GSH levels. (**h**) DCFH-DA staining for intracellular ROS. (**i**) Flow cytometric analysis of intracellular ROS levels. Fluorescence images of CT26 cells with different treatments by staining with (**j**) JC-1 (MMP), (**k**) MitoTracker Red (mitochondria), and (**l**) cleaved caspase-3 (immunofluorescence staining) and (**m**) the corresponding quantified data. (**n**) Mechanistic overview of the transformation of CuSe into CuSSe NPs by H_2_S scavenging and triggered mitochondrial impairment, caspase-3 activation, and apoptosis by enhanced PTT, CDT, and PCT effects. Scale bars 50 μm; mean ± SD; *n* ≥ 6. *P < 0.05, **P < 0.01, ***P < 0.001

The efficacy of CuSe NPs in the in vitro treatment was assessed using murine CT26 colon cancer cells. CuSe NPs were labeled with Dil as a fluorescence probe and the cellular uptake study was performed by measuring the fluorescence intensity of DiI in red within the cells. The cellular internalization of CuSe@Dil by the cancer cells via endocytosis showed prominent fluorescence intensity within 2 h after the treatment and the signal reached a maximum over 6 h (Fig. S4). The following in vitro studies were thus performed with the co-incubation with NPs for 6 h. In vitro anticancer effect of the combined PTT/CDT/PCT from the CuSe NPs against CT26 cancer cells was evaluated by 3-[4,5-dimethylthiazol-2-yl]-2,5 diphenyl tetrazolium bromide (MTT) assay where viable cells reduce the yellow MTT to purple formazan crystals via mitochondrial and cytosolic reductases and the results are quantified with the absorbance at 570 nm after dissolving the crystals [47]. The dark cytotoxicity of the NPs was increased with the concentration in the absence of the NIR irradiation (Fig. 5a). This is ascribed to the toxic effect of •OH radicals as CDT. Upon NIR irradiation, a significant enhancement in cell death, for example, with an increase of about 20% at the NP concentration of 125 µg/mL, was observed. This is attributed to the additional PTT in a concentration-dependent manner. The underlying mechanism of CDT is primarily attributed to the Fenton-like reaction, wherein Cu^+^ ions react with intracellular H_2_O_2_ to generate highly toxic •OH radicals, thereby inducing oxidative stress in H_2_O_2_ overexpressing cancer cells. It has been reported that the elevated levels of H_2_S and GSH can act as efficient ROS scavengers, thereby contributing to the resistance against oxidative damage. Given the established in situ depletion of GSH and H₂S by CuSe NPs (Fig. 4k), the NP system was further investigated in vitro to assess its capability to sensitize cancer cells to ROS-mediated therapies. First, the therapeutic effect of CuSe NPs was analyzed by MTT assay where the experiments were conducted with the cells only, NIR, H_2_S, H_2_S + H_2_O_2_, NPs, NPs + NIR, NPs + H_2_S + NIR, NPs + H_2_S + H_2_O_2_, and NPs + H_2_S + H_2_O_2_+NIR groups, with the NP concentration being preset at 75 µg/mL. To mimic TME in vitro, the experiments were carried out with an external supply of H_2_O_2_ (100 µM) and/or H_2_S (Na_2_S). Since H_2_S can act as a double-edged sword on tumor cells, being toxic against cells at high concentrations and beneficial to cell proliferation at low and moderate levels [48]. MTT assay was thus preliminarily conducted to evaluate the dependence of cellular activity on the H_2_S concentration. It was observed that the H_2_S concentrations up to 200 µM exhibited a cell proliferative effect, while the concentrations higher than that were found to be progressively toxic (Fig. S5). Thus, an H_2_S concentration of 200 µM was adopted for the entire study. The external additions of both H_2_O_2_ and H_2_S did not reduce cell viability, but otherwise showed a slight increase, indicating the antioxidant potential of H_2_S (Fig. 5b). The cell viability observed by a reduction to 63% with the NPs treatment (75 µg/mL) was further decreased to about 43% following additional NIR irradiation. With either H_2_S or H_2_O_2_ being introduced alongside the combined treatment of NPs and NIR radiation (i.e., NPs + H_2_S + NIR and NPs + H_2_O_2_ + NIR), the therapeutic effect on killing cancer cells was further enhanced. In the case of the NPs + H_2_S + NIR treatment, cell viability was reduced to about 34%, highlighting a prominent cytotoxic effect by enhanced PTT and ROS levels as a result of the transformation of CuSe into CuSSe NPs upon exposure to H_2_S. Similarly, in the NPs + H_2_O_2_ + NIR group, cell viability was reduced presumably because of the depletion of GSH in tumor cells and the increase of ROS production (via Fenton-like reactions), thereby enhancing CDT efficacy. The most profound therapeutic effect was attained with 23% in cell survival after the combined treatment of NPs and NIR irradiation with the elevated levels of H_2_S and H_2_O_2_ mimicking TME. This is ascribed to the multimodality treatment combining PTT, CDT, and PCT of the NPs along with the depletion of H_2_S and GSH. Obviously, the in situ transformation of CuSe NPs by H_2_S into CuSSe NPs plays an important contributing factor, accounting for the enhanced effects of PTT and PCT to kill cancer cells. The cell apoptosis detection by Annexin V-FITC and propidium iodide (PI) staining with flow cytometry showed a significant increase in the early apoptotic cell population with the NP treatment compared to the control groups. Moreover, the late apoptotic cell population was profoundly increased (65%) in NPs + H_2_S + H_2_O_2_+NIR group, confirming improved therapeutic efficacy (Fig. S6). The in vitro therapeutic effect was further examined by the live-dead assay. As seen in Fig. 5c, the maximum cell death was attained in the NPs + H_2_S + H_2_O_2_+NIR group, where the highest intensity of PI fluorescence signal highlighted in red was detected, in agreement with the cell viability data obtained by MTT assay. Moreover, the NPs + H_2_S + NIR group exhibited higher cytotoxicity compared to the NPs + NIR treatment. Based on the enhanced photothermal property of CuSSe NPs, CT26 cells were treated separately with CuSe NPs alone and with NPs + H_2_S, followed by NIR irradiation for 10 min. A significantly higher temperature (50 °C) with the presence of H_2_S was observed compared to merely 45 °C without H_2_S (Fig. S7a and b), being consistent with the vacancy-driven improvement in light absorption and photothermal conversion. These data elicit the synergistic performance of photocatalytic ROS generation and increased photothermal effect through in situ S doping in CuSe crystal structure and the resultant modulation of the band gap as aforementioned. In addition to the enhanced cell proliferation, it has also been reported that the overexpressed endogenous H_2_S level in colon cancer promotes migration and metastasis of cancer cells [49]. The migration of CT26 cancer cells receiving various treatments at the H_2_S concentration of 200 µM was examined by the wound healing assay. As shown in Fig. S8a, exposure to H_2_S (200 µM) effectively promotes cell migration, yet the increase in mobility is considerably attenuated for the cells further receiving CuSe NP treatment. Consistent with these findings, immunofluorescence staining of epithelial–mesenchymal transition (EMT) markers revealed that the cells and cells + H_2_S groups displayed strong vimentin expression and markedly reduced E-cadherin levels, indicating activation of the EMT program and enhanced metastatic potential. In contrast, the NPs and NPs + H_2_S groups showed suppressed vimentin expression together with restored E-cadherin levels, demonstrating that CuSe NPs effectively inhibit the H_2_S-induced EMT transition (Fig. S8b-e). The NPs-treated cells with NIR irradiation were not included in the migration assay because of significant cell death prior to the measurement. Since H_2_S can react with CuSe NPs into CuSSe NPs and Cu^2+^ can oxidize GSH to GSSG, the depletion of the intracellular antioxidant system, including GSH and H_2_S by CuSe NPs, was examined. The intracellular H_2_S and GSH levels were ascertained with Washington State Probe-1 (WSP-1) and Thioltracker violet, respectively. The images and quantified data are illustrated in Fig. 5d-g. In the positive control groups of H_2_S and H_2_S + NIR, increased WSP-1 fluorescence intensity was observed compared to the cell-only group, confirming the enhanced H_2_S level within the cells by the addition of Na_2_S. With the external supply of Na_2_S, the fluorescence intensity was significantly reduced by the treatment of CuSe NPs while a negligible difference was noted by additional NIR irradiation (Fig. 5d and S9a). The flow cytometry data in Fig. 5e also confirm H_2_S depletion by a significant reduction in WSP-1 intensity in NPs treated groups as compared to cells only and cells + H_2_S groups. The results strongly confirm the excellent H_2_S scavenging property of the CuSe NPs by the mechanism operating through direct reaction of H_2_S with CuSe NPs, irrespective of the photoactivation. Similarly, following the treatment with CuSe NPs, the cells exhibit a considerable reduction in intracellular GSH level (Fig. 5f and S9b). The reduction in the intracellular GSH level was further confirmed by the decrease in intensity in NPs treated groups as assessed by flow cytometry measurements (Fig. 5g). It is attributed to the effective oxidation of GSH by Cu^2+^ ions released from CuSe NPs, resulting in the GSH depletion and the accumulation of ROS in CT26 cells. Intracellular ROS level was examined by fluorescence staining with 2′,7′-dichlorodihydrofluorescein diacetate (DCFH-DA). The treatments were divided into six groups: namely, Cells only, H_2_O_2_ + NIR, NPs, NPs + NIR, NPs + H_2_O_2_, and NPs + H_2_O_2_ + NIR. (CuSe NPs 75 µg/mL and H_2_O_2_ 100 µM). As shown in Fig. 5h, appreciable increases in green fluorescence intensity from the cell-only group to the NPs group and further to the NPs + H_2_O_2_ group were observed, consistent with the postulation for the ROS generation via Fenton-like reaction. Despite the absence of exogenous H_2_S supply, the ROS production by the treatment with NPs + NIR as compared to only NPs was increased primarily because of the photocatalytic reduction of oxygen (to produce superoxide radicals) with the NIR irradiation on endogenous H_2_S converted CuSSe NPs. As expected, the NPs + H_2_O_2_ + NIR treatment which combines chemodynamic and photocatalytic reactions showed the highest ROS level among all other treatments. A similar trend in increases in the DCFH-DA fluorescence intensity was observed from the flow cytometry measurements of CT26 cells after the treatments, as illustrated in Fig. 5i. The data suggest that the depletion of H_2_S and GSH and the in situ transformation of CuSe NPs by endogenous H_2_S to CuSSe NPs be highly involved in the promotion of intracellular ROS level (Fig. S9c). To further delineate different ROS involved, O_2_^•−^ radical specific fluorescent dye dihydroethidium (DHE) and •OH radical specific fluorescent probe hydroxyphenyl fluorescein (HPF) were used. The flow cytometric DHE profiles (Fig. S10a) revealed pronounced increases in O_2_^•−^ production in both the NPs + H_2_S + NIR and NPs + NIR groups compared to those in the absence of NIR irradiation, supporting the photocatalytic reduction pathway inferred from the DCFH-DA data. Similarly, HPF staining (Fig. S10b) demonstrated a strong elevation of •OH levels in the NPs + H_2_O_2_ and NPs + H_2_O_2_ + NIR groups, confirming the Fenton-like generation of •OH radicals from the oxidation of Cu⁺ in the presence of H_2_O_2_. The effect of ROS on mitochondrial membrane potential (MMP) was assessed with JC-1. The results are illustrated in Fig. 5j. The cancer cells in the absence of treatment showed strong red fluorescence as a measure of normal mitochondria. Nevertheless, gradual increases in fluorescence intensity of JC-1 monomers in green as an indication of mitochondrial membrane depolarization were observed with the cells receiving treatments of CuSe NPs, NPs + NIR (or NPs + H_2_O_2_), and CuSe NPs + H_2_O_2_ + NIR in order. The density of mitochondria was examined by staining with MitoTracker Red. The probe selectively accumulates in mitochondria with an intact membrane potential, allowing visualization of normal mitochondria, but fails to label depolarized membranes due to the loss of membrane potential [50, 51]. In compliance with the JC-1 data, the fluorescence intensity from MitoTracker Red was gradually decreased with the treatments involving CuSe NPs and NIR irradiation, providing evidence to support mitochondrial dysfunction (Fig. 5k). This disruption of mitochondrial membrane potential triggers the release of cytochrome c into cytosol, binding with Apaf-1 to form the apoptosomes. These complexes then activate caspase-9, which cleaves and activates caspase-3, a critical executioner caspase responsible for driving programmed cell death [52]. Cleaved caspase-3 expression was examined to confirm apoptosis via immunofluorescence staining assay. As shown in Fig. 5l and m, the green fluorescence signal was observed in the CuSe NPs group and gradually increased in the NPs + NIR, NPs + H_2_O_2,_ and NPs + H_2_O_2_ + NIR groups, being consistent with the results illustrated in Fig. 5j and k. Consistent with the immunofluorescence staining results, Western blot analysis further confirmed a significant increase in the expression level of cleaved caspase-3 in the NP-treated groups, indicating an activation of the apoptotic cascade. Additionally, the expression levels of Bax and Bcl-2 were assessed by Western blot to evaluate mitochondrial-mediated apoptosis. The NP treatment groups showed upregulation of the pro-apoptotic protein Bax, meanwhile suppressing the anti-apoptotic protein Bcl-2 (Fig. S11). These results collectively corroborate that the NP treatment induces apoptosis through both caspase-dependent and mitochondrial pathways. Figure 5n schematically depicts the operating mechanisms involved in colon cancer cell death by CuSe NPs. Taken together, the in vitro results demonstrate that CuSe NPs effectively disrupt the antioxidant defense system while generating elevated levels of ROS, showing their promising potential as a drug-free cancer nanomedicine against CRC.

### In vivo therapeutic efficacy of CuSe NPs

**Fig. 6:**
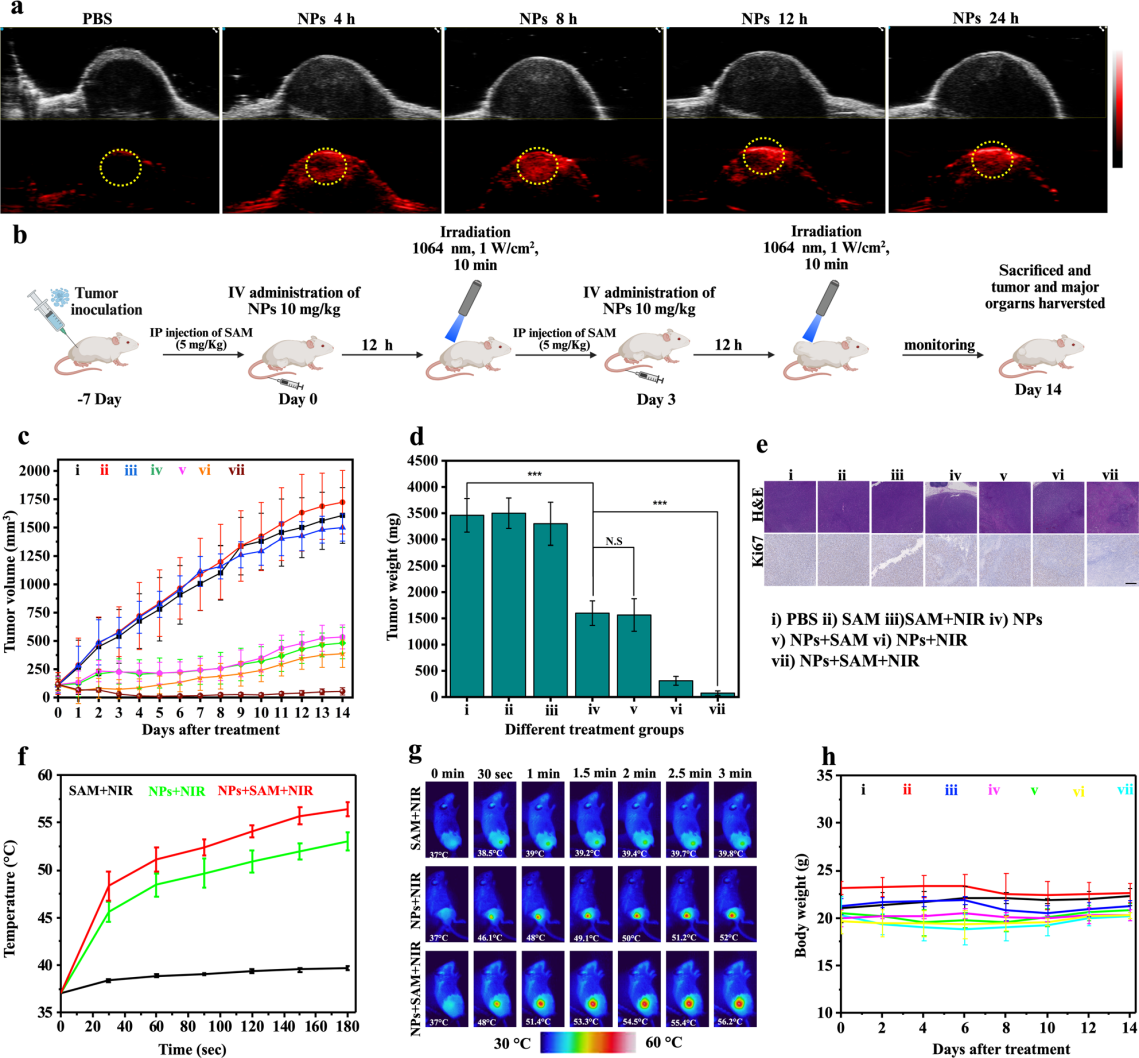
(**a**) In vivo PA images of tumors at different time points after IV administration of NPs. The yellow circle represents the inner tumor region from which the quantitative data were acquired. (b) Schematic illustration of the treatments of mice bearing subcutaneous colon cancer with NPs of two doses on day 0 and 3. (**c**) Tumor growth profiles with different treatments. (**d**) Tumor weights attained at the end of the treatments. (**e**) H&E and Ki67 staining of tumor tissues harvested at the end of the treatments. (**f**) Temperature profiles of tumors after NIR irritation in different treatment groups. (**g**) IR thermal images of tumors receiving varying treatments with NIR irradiation as captured by thermal camera. (**h**) Body weights of mice with time in different treatment groups. *P < 0.05, **P < 0.01, ***P < 0.001. *n* = 6

With the in vitro characterization of CuSe NPs against CT26 cancer cells corroborating the effect of combined PTT/CDT/PCT, the in vivo anti-tumor efficacy was studied on the mice bearing subcutaneous CT26 tumor. Male BALB/c mice (5 weeks old) were obtained from the National Laboratory Animal Center, Taiwan. All animal procedures were reviewed and approved by the Institutional Animal Care and Use Committee (IACUC) of National Tsing Hua University, Taiwan (Approval No. IACUC 113002) and carried out in accordance with established ethical guidelines for laboratory animal care. The subcutaneous CT26 tumor model was established by injecting CT26 cancer cells (3 × 10^5^/100 µL) subcutaneously into the right flank of each mouse. To evaluate the in vivo biodistribution, the mouse serum albumin (MSA) coated NPs (10 mg/kg) were administered intravenously once tumors reached an approximate volume of 100 mm^3^. The accumulation of the NPs in tumor and major organs was quantified at various time points after tail vein injection using inductively coupled plasma mass spectrometry (ICP-MS). The results as shown in Fig. S12a revealed progressive accumulation of NPs in tumor with a peak concentration observed at 12 h post injection. Fig. S12b clearly illustrates a substantial accumulation of CuSe NPs in tumor compared to that in major organs, most likely because of the enhanced permeability and retention effects. A relatively high level of NPs was also observed in liver, being consistent with the uptake by the reticuloendothelial system, a common fate for intravenously administered NPs. The accumulation of the NPs in tumor was also corroborated using photoacoustic (PA) imaging system (Vevo LAZR-X) and the data are presented in Fig. 6a. The accommodation of NPs in tumor region was clearly observed along with the highest tumor signal intensity occurring at 12 h after the administration (Fig. S13), in agreement with the data attained by ICP-MS.

To assess the therapeutic efficacy of MSA coated CuSe NPs in vivo, treatment studies were carried out in mice bearing subcutaneous CT26 colon tumors. With the tumor size reaching 100 mm^3^, the animals were randomly assigned to seven experimental groups (PBS, SAM, SAM + NIR, NPs, NPs + SAM, NPs + NIR, and NPs + SAM + NIR), with six mice in each group (*n* = 6). S-adenosyl-l-methionine (SAM), a known enhancer of CBS enzymatic activity, was intraperitoneally administered 12 h prior to NP injection in NPs + SAM and NPs + SAM + NIR groups to modulate endogenous H_2_S levels [53]. CuSe NPs were IV administered at a dose of 10 mg/kg on day 0 and 3. In groups receiving photothermal treatment, NIR laser irradiation at 1064 nm was applied for 10 min at 12 h post-injection (Fig. 6b). Following intraperitoneal injection and NIR laser irradiation, the tumor volumes of mice receiving free SAM and SAM + NIR treatments increased significantly with time, similar to that of the PBS group (Fig. 6c). In contrast, the treatment with CuSe NPs alone considerably suppressed tumor growth, achieving approximately a one-third reduction in tumor volume compared to the control groups within the first 7 days. Importantly, the NPs + SAM group exhibited a similar level of tumor suppression to that of NPs only, indicating that the elevated H_2_S level induced by SAM did not accelerate tumor progression. This outcome supports the notion that CuSe NPs effectively scavenge endogenous H_2_S in vivo, converting themselves into CuSSe and maintaining therapeutic efficacy. Further enhancement in tumor inhibition was observed in the NPs + NIR group, where the combination of PTT and CDT yielded enhanced antitumor effect during the early phase of treatment. Nevertheless, tumor regrowth occurred by day 14, with sizes reaching ~ 400 mm^3^. By contrast, the NPs + SAM + NIR group demonstrated the most potent therapeutic effect, dramatically reducing tumor size and showing complete regression in several cases. By day 14, the average tumor volume was approximately 50 mm^3^ with no evidence of recurrence. The mean tumor weights (Fig. 6d) of tumors harvested on day 14 from different treatments are consistent with the tumor growth profiles illustrated in Fig. 6c, further validating the therapeutic outcomes. While immune cell infiltration was not directly assessed in this study, the superior therapeutic efficacy of the NPs + SAM + NIR treatment can be rationalized by the synergistic effects introduced through band-gap modulation. Sulfidation driven band gap modulation enhances both PCT and PTT performance while simultaneously depleting intratumoral H_2_S, thereby increasing the oxidative stress. The resulting elevation in ROS not only mediates direct cytotoxicity but is also known to trigger immunogenic cell death pathways [54]. In particular, the release of damage-associated molecular patterns, including calreticulin, HMGB1, ATP, and heat-shock proteins, can promote dendritic cell maturation and antigen presentation. Matured dendritic cells facilitate the transformation of natural T cells into cytotoxic T lymphocytes, amplifying adaptive immune responses [55].

The histological examination of tumor tissues receiving NPs + SAM + NIR treatments by hematoxylin and eosin (H&E) staining revealed substantial tissue damage, attributed primarily to cell apoptosis. By contrast, the tissue damage was somewhat attenuated in tumors treated with NPs and either laser irradiation or SAM pretreatment. Additionally, Ki67 staining showed a reduced number of Ki67-positive cells in the NPs treated groups, the NPs + SAM + NIR group being the least compared to other groups, indicating effective suppression in tumor cell proliferation (Fig. 6e).

Tumor temperature was recorded using an IR thermal camera during NIR laser exposure. The corresponding thermal profiles and IR images are presented in Fig. 6f and g, respectively. A significant increase in tumor temperature was observed, reaching approximately 52 °C in the groups treated with CuSe NPs and NIR irradiation. The most pronounced hyperthermic effect occurred in the NPs + SAM + NIR group, where the tumor temperature reached up to 56 °C, indicating that SAM effectively enhanced the CBS enzyme activity and thus the H_2_S levels in tumor. This facilitates the in situ formation of CuSSe NPs in company with superior photothermal performance in the NIR-II window. Immunofluorescence staining of heat shock protein 70 (HSP70) revealed an elevated heat shock response in both NPs + NIR and NPs + SAM + NIR tumors, consistent with the high intratumoral temperatures achieved during PTT (Fig S14). Notably, despite comparable HSP70 upregulation, only the NPs + SAM + NIR group exhibited sustained tumor suppression without recurrence. While HSP70 upregulation is a common cellular response to thermal stress [56], the enhanced therapeutic outcome in the NPs + SAM + NIR group is likely driven by the combined effects of higher photothermal temperature and ROS produced by CDT and PCT, which together overcome heat-induced cytoprotective mechanisms. Throughout the 14-day treatment period, no significant changes in body weight were observed in all the groups, indicating that CuSe NPs did not cause acute systemic toxicity (Fig. 6h). To further investigate the antitumor effects of CuSe NPs, fluorescence staining was performed on tumor tissue sections. DCFH-DA staining on ROS revealed a clear increase in fluorescence intensity (in green) in the tumor tissues of the NP-treated group, confirming the promotion in ROS production (Fig. S15). The NIR-treated groups showed further enhancement in ROS levels due to the photocatalytic reduction of O_2_ to O_2_^•−^ radicals. ROS induces DNA damage through DNA strand breakage. TUNEL assay showed the highest percentage of DNA damage in the NPs + SAM + NIR group (Fig. S15), indicating the greatest extent of apoptotic cell death in this treatment cohort.

**Fig. 7:**
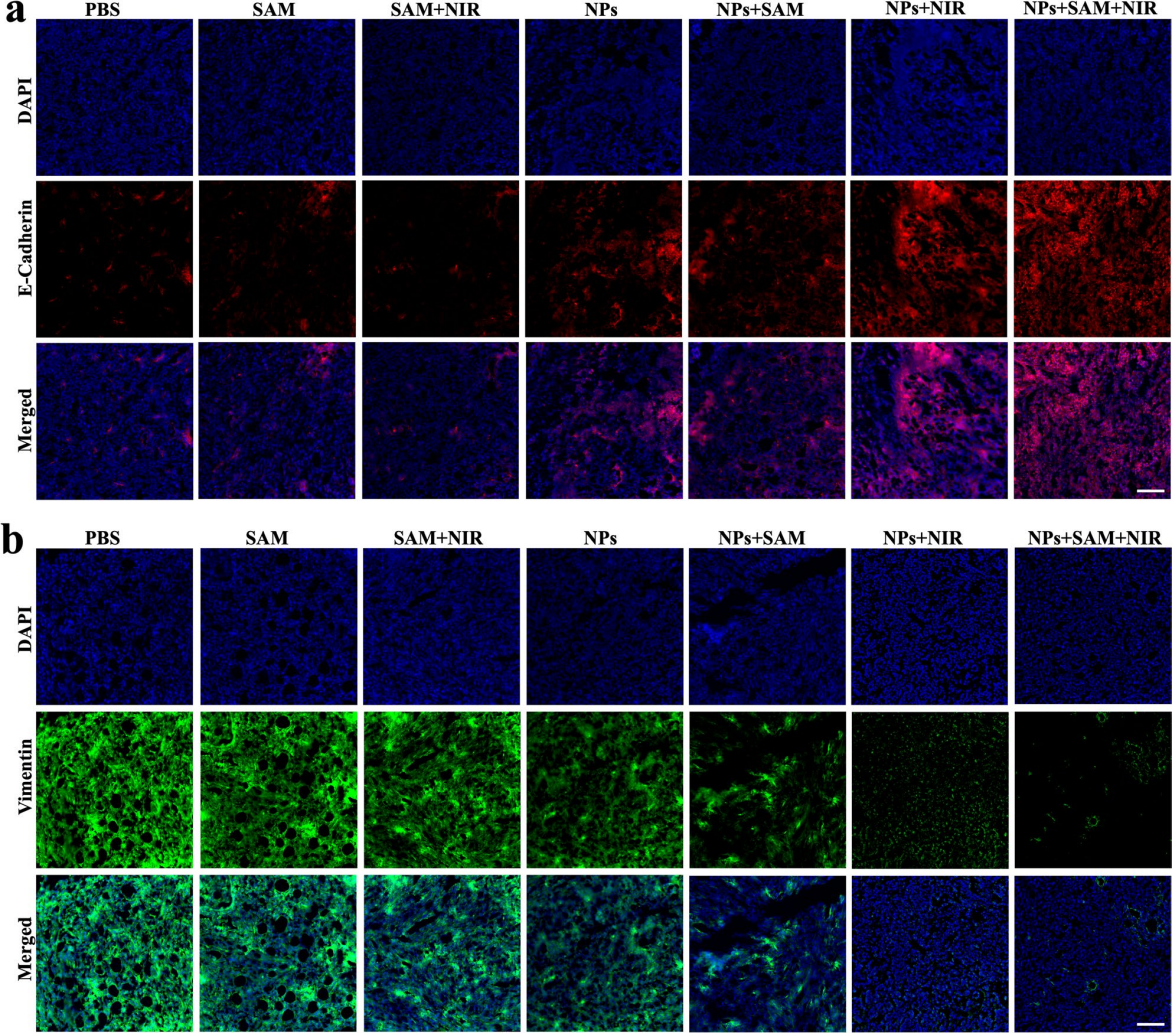
Immunofluorescence staining analysis of (**a**) E-cadherin (red) and (**b**) vimentin (green) expression in tumor tissues after different treatments. Scale bar 100 μm

H_2_S depletion plays a critical role in tumor growth suppression, as elevated H_2_S levels promote cellular proliferation and angiogenesis, which facilitate tumor expansion [4, 6]. CuSe NPs effectively scavenge H_2_S, disrupting these processes and reprogramming a TME less favorable for tumor survival. This was demonstrated by the reduced fluorescence intensity of WSP-1 in tumor sections from the NP-treated groups, confirming the H_2_S-scavenging capability of CuSe NPs (Fig. S16a). To assess the impact on angiogenesis, CD31 expression, a marker of endothelial cells and vascular development, was evaluated. While high CD31 expression was observed in the SAM and SAM + NIR groups, immunofluorescence staining on CD31 in tumor tissues receiving NP treatments showed significant reduction in the CD31 level. (Fig. S16b). To further evaluate the metastatic phenotype in vivo, tumor sections were stained for E-cadherin and vimentin. Control tumors displayed high vimentin expression and reduced E-cadherin expression, indicating activation of the EMT. In contrast, NPs-treated tumors showed restored E-cadherin and suppressed vimentin levels (Fig. 7a, b), demonstrating that CuSe NPs effectively inhibit EMT progression in vivo. Together, the reduced CD31 levels and the restored E-cadherin with suppressed vimentin expression confirm that the NP treatment simultaneously inhibits angiogenesis and reverses the EMT program, thereby suppressing metastatic progression. In parallel, the shift in band potentials induced by sulfidation enables CuSSe NPs to catalyze the oxidation of H_2_O_2_ under NIR irradiation, thereby generating O_2_ that alleviates tumor hypoxia and downregulates HIF-1α expression. Strong HIF-1α fluorescence was detected in both the control and NPs alone groups, indicating substantial presence of tumor hypoxia. In contrast, markedly reduced HIF-1α signal was observed in the NPs + NIR and NPs + SAM + NIR groups, reflecting effective hypoxia alleviation via photocatalytic O_2_ generation (Fig. S17). In this study, the results suggest that CuSe NPs inhibit tumor growth through a combination of CDT, PCT and PTT, while also limiting cell proliferation by scavenging excess H_2_S in TME.

**Fig. 8:**
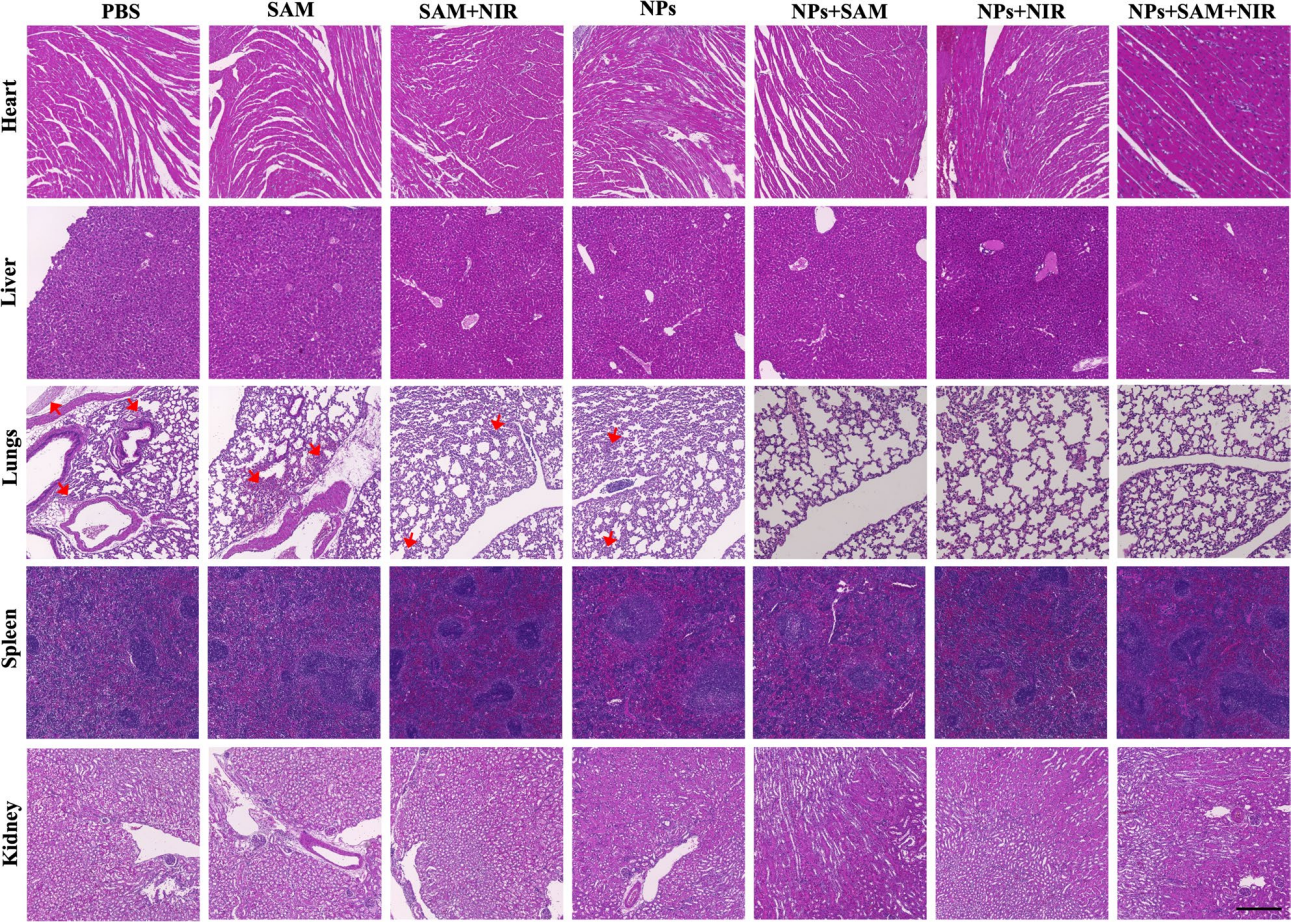
H&E tissue staining of major organs after different treatments. Red arrows indicate the metastatic region in lungs. Scale bar 200 μm

The histological examination of major organ tissues indicated no discernible organ damage, indicating the excellent biosafety of the NP doses administered intravenously in this study (Fig. 8). The H&E staining provides direct evidence that the CuSe NPs not only suppress primary tumor burden but also limit early metastatic dissemination, as reflected by barely detected pulmonary micrometastases in lungs in the NP treated groups compared with the clear micrometastatic foci observed in control groups. The hemolysis tests were conducted with MSA-coated CuSe NPs of various concentrations. The results demonstrated that the hemolysis percentage remained below 5% for the NP concentrations up to 200 µg/mL, indicating minimal toxicity to red blood cells and confirming the hemocompatibility of the NPs in the concentration range employed (Fig. S18). The blood biochemistry analyses focusing on liver and kidney functions also demonstrated negligible toxicity across all treatment groups (Fig. S19). The H&E examination, hemolysis tests, and blood biochemistry assay strongly support the commendable biological safety profile of the treatment throughout the study.

## Conclusions

In this study, a NIR-II responsive plasmonic semiconductor NP system as a multifunctional hybrid nanotherapeutics was developed for colon cancer treatment. The semiconductor NPs exhibited hexagonal morphology, playing the roles as plasmon and semiconductor nanotherapeutics to achieve photothermal response to NIR-II irradiation. The CuSe NPs underwent endogenous H_2_S depletion in the H_2_S-rich microenvironment of CRC whereby CuSSe NPs were thus formed in situ. This led to the modulation of the bandgap of the NPs, enhancing their photothermal performance and ability to generate ROS by oxygen reduction with exposure to NIR-II laser irradiation in addition to the hydroxy radical generation by Cu^2+^ with H_2_O_2_ following Fenton-like reaction. Thus, the improved PTT/CDT/PCT mediated by albumin-coated CuSe NPs demonstrated potent cytotoxicity in both in vitro and in vivo characterization against colon tumor. The in vitro data revealed that CuSe NPs effectively induced apoptosis in CT26 colon cancer cells. The in vivo results manifested the successful suppression of colon cancer in subcutaneous tumor bearing mice following the treatment with CuSe NPs by intravenous administration and NIR-II irradiation at 1064 nm. The blood biochemistry assay, body weight measurements, hemolysis examination, and H&E staining of major organ tissues signified the excellent biocompatible nature of the CuSe NPs. Importantly, this study paved the way toward photocatalytic cancer therapy through the in vivo band gap modulation of semiconductor crystal structure.

## Materials and methods

### Materials

Copper sulfate pentahydrate (CuSO_4_.5H_2_O), selenium dioxide (SeO_2_), sodium borohydride (NaBH_4_), PVA, BSA, MSA, DMPO, sodium sulfide nonahydrate (Na_2_S·9H_2_O), SAM, MTT, 5,5′-dithiobis-2-nitrobenzoic acid (DNTB), DCFH-DA, N,N-dimethyl-p-phenylenediamine (DMPD), DPBF, 5,5-dithio-bis-(2-nitrobenzoic acid (DTNB), and WSP-1 were purchased from Sigma-Aldrich (USA). RPMI 1640 medium, fetal bovine serum (FBS), 0.25% trypsin–EDTA solution, and penicillin–streptomycin solution were obtained from Thermo Fisher Scientific (Waltham, MA, USA). All other chemicals were of reagent grade and used as received. CT26 cells (murine colon adenocarcinoma cell line) were purchased from the Food Industry Research and Development Institute, Hsinchu City, Taiwan. The male BALB/C mice aged 6 weeks were obtained from the National Laboratory Animal Center in Taiwan. The procedures approved by the IACUC: 113002 for handling and using laboratory animals were strictly followed during the experiments.

### Syntheses of cuse and CuSe@albumin NPs

CuSe NPs were synthesized by a hydrothermal method. In brief, an aqueous solution of PVA (500 µL, 10 mg/mL) was separately mixed with CuSO_4_ (500 µL, 50 mM) and SeO_2_ (500 µL, 50 mM). An aqueous solution of NaBH_4_ (500 mM) freshly prepared was added separately to the precursor solutions under stirring at room temperature. The SeO_2_ and CuSO_4_ solutions were added to a PTFE-lined reaction chamber containing DI water (9.6 mL) under vigorous stirring. The reaction was conducted in a Teflon-lined autoclave at 100 °C for 12 h. After cooling to room temperature, CuSe NPs were collected by centrifugation at 6000 rpm, washed with DI water repeatedly to remove unreacted residues, and stored at 4 °C until further use. To prepare albumin-coated NPs, the CuSe NPs and albumin (either BSA or MSA) were mixed at a weight ratio of 4:1 in phosphate buffer (10 mM, pH 7.4). The mixture was stirred gently at ambient temperature for 6 h. The resulting CuSe@albumin NPs were collected by centrifugation at 4500 rpm, washed twice with DI water to remove unbound BSA (or MSA), and stored at 4 °C for further use.

### Morphologic and structural characterization of CuSe and CuSSe NPs

The size and zeta potential measurements of the CuSe NPs were carried out with DLS on a ZetaSizer Nano Series instrument from Malvern. The size and morphology of CuSe NPs before and after sulfidation were analyzed with TEM (JEOL JEM-2100 F, Japan) and HRTEM (JEOL JEM-F200, Japan). Crystal structure examination of the NPs was performed on an XRD, BRUKER D8. The absorption spectra of NPs were obtained on a U2900, Hitachi UV – Vis spectrometer. DRS examination of CuSe and CuSSe NPs was measured by UV/Vis spectrophotometer (Hitachi U-4100). ROS was identified with EPR spectroscopy (BRUKER, ELEXSYS, E-580). The elemental compositions and oxidation state of CuSe NPs were examined by XPS (ULVAC-PHI, PHI Quantera II, beam: 6 mA, voltage: 12 kV, energy standard: C 1s = 284.80 eV). VB potential was determined using high-resolution XPS (ULVAC-PHI). The photothermal performance was evaluated upon exposure to NIR-II irradiation at 1064 nm with an IR thermal camera (Thermo Shot F20, NEC Avio Infrared Technologies, Germany).

### Stimuli-responsive performance of CuSe NPs

#### Electronic band structure evaluation

The optical band gaps of CuSe and CuSSe NPs were evaluated with DRS. The reflectance data (R) obtained by DRS were used to calculate the Kubelka–Munk function defined as F(R)=(1 − R)^2^/2R. The optical band gap was estimated by plotting [F(R)⋅h ν]^2^ vs. photon energy (eV) with extrapolation in the linear region of the Tauc plot to the energy axis. The VB position of CuSe and CuSSe NPs was determined with XPS. The VB spectrum was recorded by scanning the energy region near the Fermi level. The VB maximum was estimated by extrapolating the linear portion of the leading edge of the VB spectrum to the baseline.

#### Photothermal performance and photothermal conversion efficiency

The photothermal performance of CuSe NPs was evaluated by monitoring the temperature change in aqueous dispersions under NIR irradiation. CuSe NP solutions of varying concentrations (25, 50, 75 and 100 µg/mL) were irradiated using 1064 nm laser (1.0 W/cm^2^) for 10 min, both before and after exposure to H_2_S (1.0 mM). The temperature changes were recorded by the infrared thermal camera. The temperature variation under different power densities (0.5, 1.0, 1.5 W/cm^2^) of 1064 nm laser and the photothermal stability of CuSe NPs (50 µg/mL) was assessed through repeated on/off laser irradiation cycles with temperature profiles captured every minute to evaluate performance consistency over time. The photothermal conversion efficiencies (η) of CuSe and CuSSe NPs were attained by NIR irradiation at 1064 nm (1.0 W/cm^2^) of the aqueous dispersion of NPs (100 µg/mL) until the temperature reached a plateau. The solution was allowed to cool down to ambient temperature naturally, with continuous monitoring of the temperature-time profile. The efficiency was calculated as follows.

$$\:\eta\:=\frac{hS\left({T}_{max}-{T}_{surr}\right)-{Q}_{dis}}{I\left(1-{10}^{-{A}_{\lambda\:}}\right)}$$

where *h* is the heat transfer coefficient, *S* the surface area of heat transfer, *T*_*max*_ the plateau temperature, *T*_*surr*_ the temperature of the surrounding environment, *Q*_*dis*_ the heat dissipated by the surrounding environment, *I* the radiation intensity of the incident light, and *A*_*λ*_ the absorbance of NPs at 1064 nm.

#### GSH depletion

The depletion of GSH by CuSe NPs was evaluated by reaction of the NPs (50 and 100 µg/mL) with GSH (5.0 mM) in phosphate buffer at 37 °C for 3 h. After the reaction, residual GSH was quantified by mixing the supernatant of the NP dispersion after centrifugation with DTNB (50 µM) in Tris buffer (pH 8.4), followed by the absorbance measurement at 412 nm by UV–Vis spectroscopy.

#### H_2_S scavenging

Methylene blue assay was used to study the H_2_S scavenging property of CuSe NPs following a previously established protocol.^[44]^ NPs of different concentrations were treated with Na_2_S (1.0 mM) in DI water for 30 min. After the reaction, the supernatant (100 µL) attained by centrifugation was mixed with zinc acetate solution (1%; 100 µL) and then added to a pre-mixed FeCl_3_/DMPD solution. Absorbance at 670 nm was measured after the reaction for 20 min using a SpectraMax M5 microplate reader.

#### ROS generation

CuSe NPs (100 µg/mL) were incubated with H_2_O_2_ (1.0 mM) for 30 min, followed by the addition of DMPO (30 µM) as a spin-trapping agent for the detection of •OH by EPR spectroscopy. In addition to EPR analysis, the generation of •OH radicals was further assessed under both dark and NIR-irradiated conditions (1064 nm, 1 W/cm^2^, 10 min) using TPA as a fluorescent probe, with quantification performed by fluorescence spectroscopy. In parallel, the production of O_2_^•−^ by CuSe and CuSSe NPs (400 µg/mL) was evaluated using DPBF as fluorescence probe and DMPO (30 µM) as a spin-trapping agent, in the presence and absence of NIR irradiation. DPBF was first dissolved in a 1:1 ratio of 99% ethanol and DMSO to prepare a 5.0 mM stock solution. For each assay, an appropriate volume of the stock was added to the aqueous NP suspension to achieve a final DPBF concentration of 20 µM. The mixture was vortexed for 10 s to ensure homogeneous distribution before irradiation.

#### O_2_ generation

Oxygen production by CuSe and CuSSe NPs was evaluated using a BANTE portable dissolved oxygen meter. NP suspensions (1 mg/mL) were prepared in water and subjected to NIR irradiation at 1064 nm for 10 min. Dissolved oxygen levels were recorded during the irradiation period. To assess the role of photogenerated holes, a parallel experiment was performed using a 1:1 methanol–water mixture as the dispersion medium, where methanol served as a hole scavenger.

### In vitro characterizations

#### Cellular uptake study

The internalization of CuSe@albumin NPs by CT26 cells was evaluated using DiI labeling. After coincubation of NPs with cells for various time intervals (2, 4, 6, and 8 h), nuclei were counterstained with Hoechst 33,342 and fluorescence images were obtained using a fluorescence cell imaging system (ImageXpress Pico, Molecular Devices, USA), and intracellular DiI fluorescence intensity was quantified with ImageJ software.

#### Cytotoxicity assay

The cytotoxicity of CuSe NPs was assessed with the standard MTT assay. First, the concentration-dependent cytotoxicity of CuSe NPs was evaluated under both dark and NIR irradiation conditions. To better understand the therapeutic mechanisms, the cytotoxicity assay was performed in various groups, where the concentrations of H_2_O_2_ and Na_2_S at 100 µM and 200 µM were used. After incubation with NPs for 6 h, cells were NIR irradiated (1064 nm, 1 W/cm^2^, 10 min) when applicable and incubated for 24 h before MTT analysis. Live/dead staining was performed using Calcein-AM and PI to visualize viable and dead cells under a fluorescence microscope. Flow cytometry measurements of live-dead staining was also performed on a Cytoflex flow cytometer (Beckman) with Annexin V-FITC and PI staining to confirm the apoptosis in CT26 cells after various treatments.

#### Intracellular H_2_S and GSH depletion

Intracellular H_2_S and GSH levels were evaluated by staining with WSP-1 and ThiolTracker™ Violet, respectively. The concentrations of CuSe NPs (75 µg/mL), Na_2_S (200 µM), and H_2_O_2_ (100 µM) were used. NIR laser irradiation at 1064 nm with the power density of 1 W/cm^2^ for 10 min was adopted where applicable. After treatments, cells were stained with WSP-1 for H_2_S identification and ThiolTracker™ Violet for GSH and images were captured with fluorescence microscopy. Hoechst 33,342 was used to stain cell nuclei, and the fluorescence intensity was quantified using ImageJ. Flow cytometry measurements were further conducted to examine the intracellular levels of GSH and H_2_S after various treatments.

#### Cell migration assay

To investigate the potential of CuSe NPs to reduce cell migration through H_2_S suppression, CT26 cells (1 × 10^5^ cells/mL) were cultured in a migration chamber (8 μm, Corning, USA) for 12 h. The cells were then treated with the NPs (50 µg/mL) and H_2_S (200 µM, 6 h). Cell migration was then assessed after 24 h and compared to the cells without NP treatment using an optical microscope (Olympus IX70).

#### Intracellular ROS detection

Intracellular ROS level in CT26 cells was evaluated with the DCFH-DA staining assay. The incubation of the cells with NPs was performed for 6 h. Immediately after NIR irradiation (1064 nm, 1 W/cm^2^, 10 min), cells were stained with DCFH-DA (50 µM) for 30 min, followed by washes with PBS and fixation with 4% formaldehyde. The fluorescence signals were captured by a fluorescence imaging system and quantified using ImageJ software. To concretely prove O_2_•⁻ and •OH generation in cells, specific probes DHE and HPF were used respectively. The intracellular ROS levels of the cells after various treatments were examined by flow cytometry.

#### Immunofluorescence analysis of EMT markers

Expression of E-cadherin and vimentin after different treatments was checked using immunofluorescence staining and quantified with ImageJ.

#### MMP examination and cleaved caspase-3 staining

JC-1 staining was performed to examine the MMP of CT26 cancer cells after various treatments (Control, H_2_O_2_ + NIR, NPs, NPs + NIR, NPs + H_2_O_2_ and NPs + H_2_O_2_ + NIR). JC-1 (1.0 µM) was used for mitochondrial membrane staining. At high MMP, JC-1 accumulates in mitochondria as aggregates emitting red fluorescence, whereas at low MMP, it remains as monomers with green fluorescence. The distribution of JC-1 in cells in a monomer or aggregate state was determined using a fluorescence imaging system. Similarly, MitoTracker deep red (1 µM), a mitochondria membrane potential-dependent probe, was also used to examine the normal mitochondria inside the cells.^[51]^ Immunofluorescence staining of cleaved caspase-3 was carried out to assess apoptotic activation. After primary and FITC-labeled secondary antibody reaction, fluorescence images were captured and quantified.

#### Western blot analysis

Cells were seeded in 6-well plates and treated with NPs for 6 h. After treatment, cells were washed with cold PBS and lysed using RIPA buffer supplemented with protease inhibitors. The lysates were centrifuged to remove cell debris and NP aggregates, and the supernatant was collected for protein quantification using a BCA assay. Equal amount of protein was mixed with loading buffer, separated by SDS-PAGE, and transferred onto nitrocellulose membrane. Membranes were then blocked using 5% BSA solution, incubated with primary antibodies against Bcl-2, Bax, cleaved caspase-3, and β-actin overnight at 4 °C. After washing of the primary antibody, HRP linked IgG for rabbit secondary antibody was added, and the images were taken using a syngene G: BOX gel and blot imaging systems. Quantitative analysis of the proteins band was performed using ImageJ.

### In vivo biodistribution, antitumor efficacy and biosafety evaluation

The antitumor efficacy and biosafety of CuSe NPs were assessed in CT26 tumor-bearing mice following the procedures reviewed and approved by IACUC of National Tsing Hua University, Taiwan (Approval No. IACUC 113002). Male BALB/c mice (6 weeks old) were subcutaneously inoculated with CT26 cells (3 × 10⁵ cells/100 µL) into the right flank. The mice were IV injected with CuSe NPs at a single dose of 10 mg/kg with the tumor size reaching about 100 mm^3^ calculated as (W^2^ × L)/2, where W and L are the tumor width and length, respectively, based on Vernier caliper measurements. PA imaging was conducted at different time points post-injection to monitor NP accumulation in tumors. For the biodistribution study, tumor-bearing mice were sacrificed at 12 h after the IV administration of NPs (single dose). The tumor and major organs (heart, liver, lung, spleen, and kidney) were harvested. The Cu levels were determined by ICP-MS (Elan DRC II, PerkinElmer SCIEX). For the antitumor efficacy evaluation, at the tumor size of about 100 mm^3^, mice were randomly divided into seven groups. Mice were treated with MSA-coated CuSe NPs of two doses (200 µL, 10 mg/kg each dose) by IV injection on day 0 and 3. It was followed by NIR irradiation (1064 nm, 1 W/cm^2^) for 10 min at 12 h after the NP administration. The local tumor temperature changes were monitored with the IR thermal imaging camera. Tumor volume and body weight were recorded every day. On day 14, mice were sacrificed, and blood samples were collected for blood biochemistry assays. Major organs were harvested for histological analysis with H&E staining. For hemocompatibility assessment, CuSe NPs (25, 50, 100, 200 µg/mL) were incubated with freshly collected mouse blood at 37 °C for 6 h, and hemolysis was determined by measuring hemoglobin release at 541 nm via UV-Vis spectroscopy. Hemolysis rate below 5% was considered acceptable for systemic administration. Tumor tissue sections were subjected to immunohistochemistry staining of Ki67 to determine cell proliferation. Intratumoral ROS levels were detected with the fluorescence probe, DCFH-DA, and H_2_S levels visualized with WSP-1. Apoptotic DNA fragmentation was examined through TUNEL staining. Tumor angiogenesis was assessed by immunofluorescence staining of CD31 as an angiogenesis marker and the level of hypoxia in tumor was evaluated by staining of HIF-1α in a similar manner.

### Statistical analysis

All measurements were performed at least in triplicate. Results were expressed as mean ± standard deviation (SD). All statistical data were processed in Origin 2022. Two groups were compared using two-sided Student’s t-tests, while comparisons among three or more groups were performed using one-way or two-way ANOVA, depending on the experimental design. A P-value less than 0.05 was considered statistically significant. The significance levels were indicated as follows: ns (no significance), *P < 0.05, **P < 0.01, ***P < 0.001.

## Supplementary Information

Below is the link to the electronic supplementary material.

Supplementary Material 1.

## Data Availability

The data that support the findings of this study are available from the corresponding author upon reasonable request.
