# Supplementary material for: H2S-responsive CuSe nanoparticles convert into CuSSe in situ with band gap modulation for oxygen self-supply and enhanced colon cancer phototherapy
Source: J Nanobiotechnology. 2025 Dec 31;24:106. doi: 10.1186/s12951-025-03977-9 (PMC12866474; doi:10.1186/s12951-025-03977-9)
Supplement: Supplementary file 1 — Supplementary Material 1. [file 12951_2025_3977_MOESM1_ESM.docx]

**Supplementary Information**

**H_2_S-Responsive CuSe Nanoparticles Convert into CuSSe in Situ with Band Gap Modulation for Oxygen Self-Supply and Enhanced Colon Cancer Phototherapy**

Manoj Kandel^1^, Arjun Sabu^1^, Kai-Min Chan^2^, Ramalingam Sharmila^1^, Lakshminarayan Ramesan^1^, Ming-Yin Shen^1,3^,* Yu-Fen Huang^1,2^,* Hsin-Cheng Chiu^1^*

^1^Department of Biomedical Engineering and Environmental Sciences, National Tsing Hua University, Hsinchu City, Taiwan 300.

^2^Institute of Analytical and Environmental Sciences, National Tsing Hua University, Hsinchu City, Taiwan 300.

^3^Department of Surgery, China Medical University Hsinchu Hospital, Hsinchu County, Taiwan 302.

*Corresponding authors at: Department of Biomedical Engineering and Environmental Sciences, National Tsing Hua University, Hsinchu 300, Taiwan. E-mail address: hscchiu@mx.nthu.edu.tw (H.-C. Chiu), yufen@mx.nthu.edu.tw (Y.-F. Huang) and mingyin.shen@gmail.com (M.-Y. Shen).

**
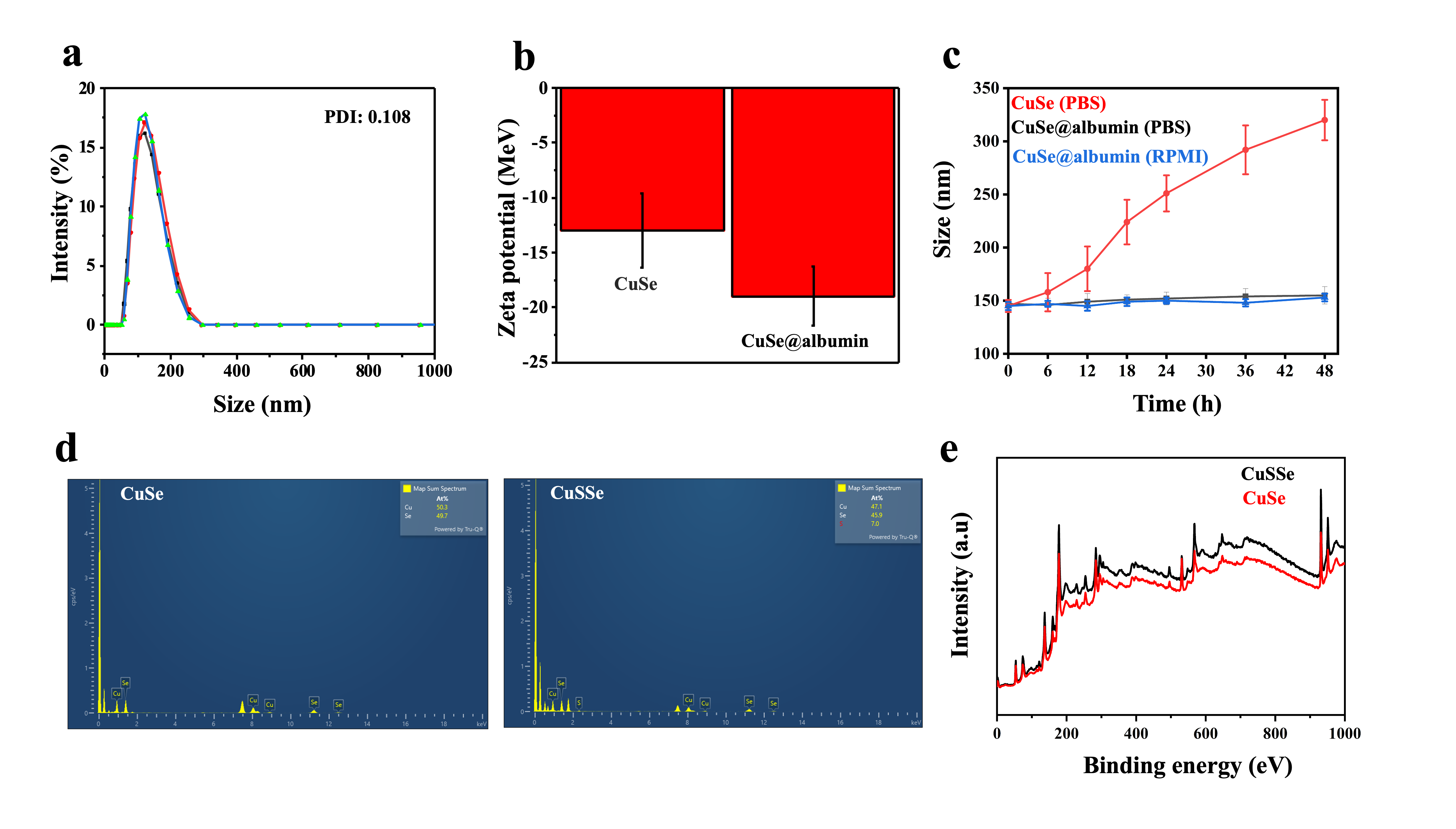
**

**Fig. S1.** (a) DLS size distribution profile of CuSe@albumin NPs in aqueous solution. (b) Zeta potentials of CuSe and CuSe@albumin NPs. (c) Colloidal stability measurements of CuSe NPs in PBS and CuSe@albumin NPs in PBS and RPMI in terms of the change in the mean hydrodynamic diameter of NPs with time. (d) EDX spectra of CuSe and CuSSe NPs. (e) XPS survey spectra of CuSe NPs before and after the reaction with H_2_S.


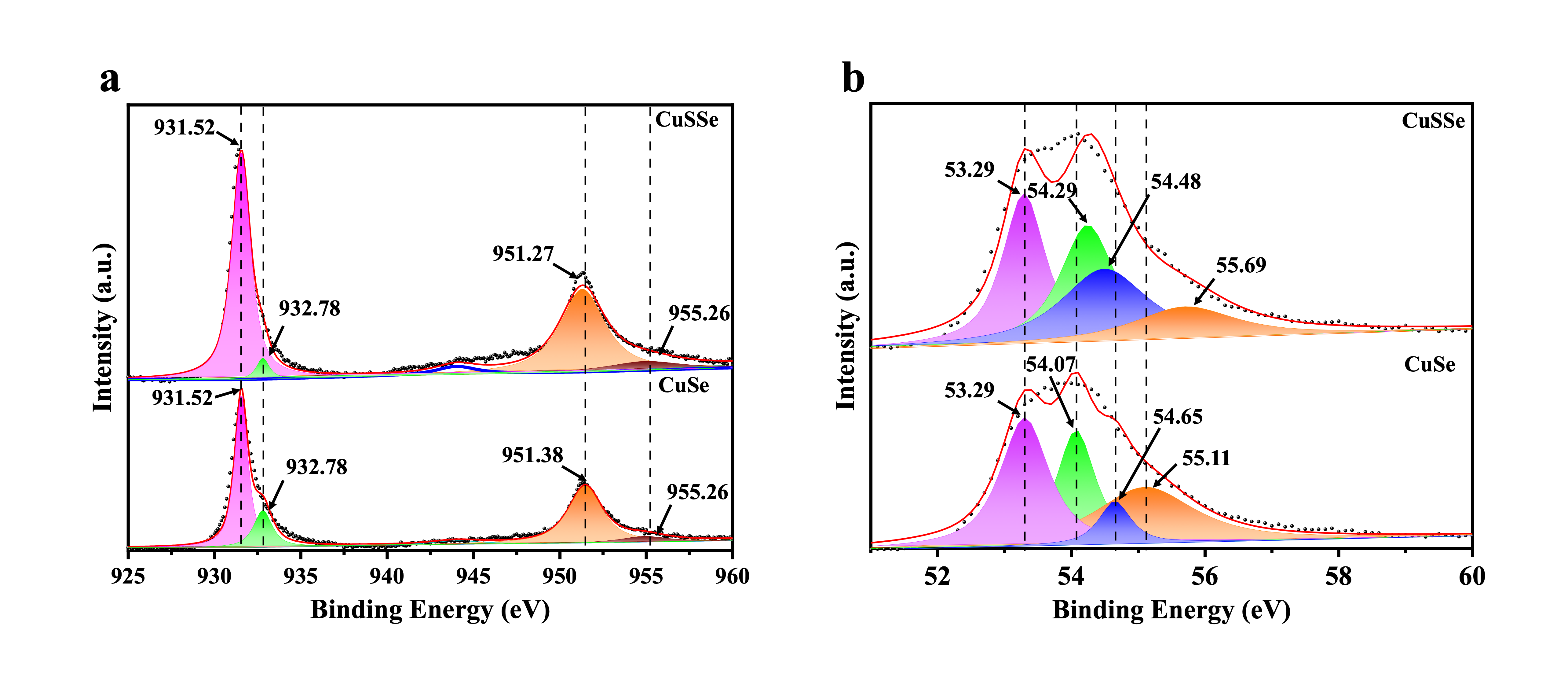


**Fig. S2.** Combined **(a)** Cu 2p and **(b)** Se 3d core-level XPS spectra of CuSe and CuSSe NPs.


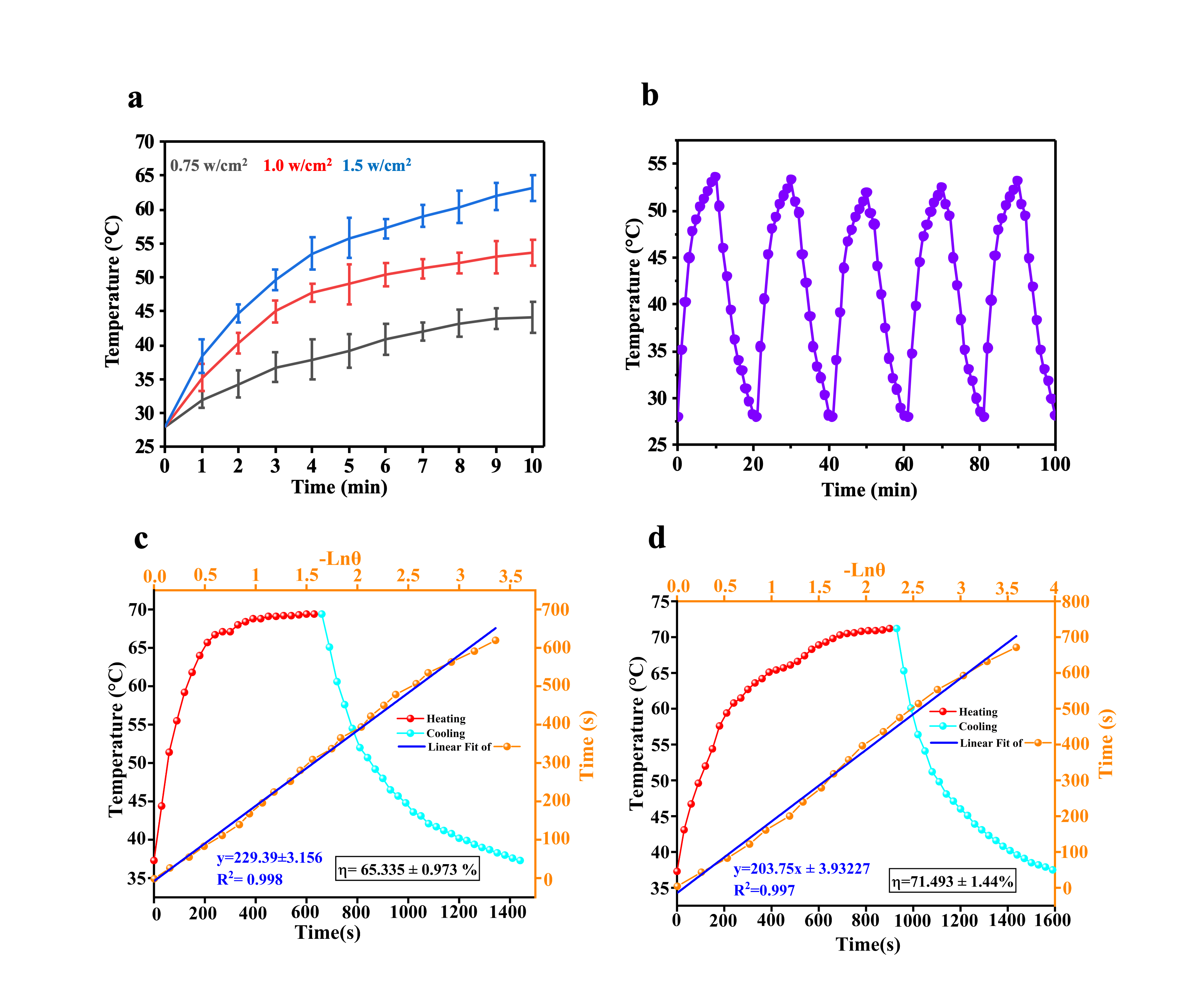


**Fig. S3.** (a) The temperature profiles of aqueous dispersions of CuSe NPs (50 μg/mL) by NIR irradiation with different laser power densities (0.5, 1.0, and 1.5 W/cm^2^). (b) The temperature profiles of CuSe NPs with repeated ON/OFF irradiation cycles, with each ON and OFF duration of 10 min for five cycles. Calculation of photothermal conversion efficiency of (c) CuSe and (d) CuSSe NPs under NIR laser irradiation (1064 nm) at a power intensity of 1.0 W/cm^2^.


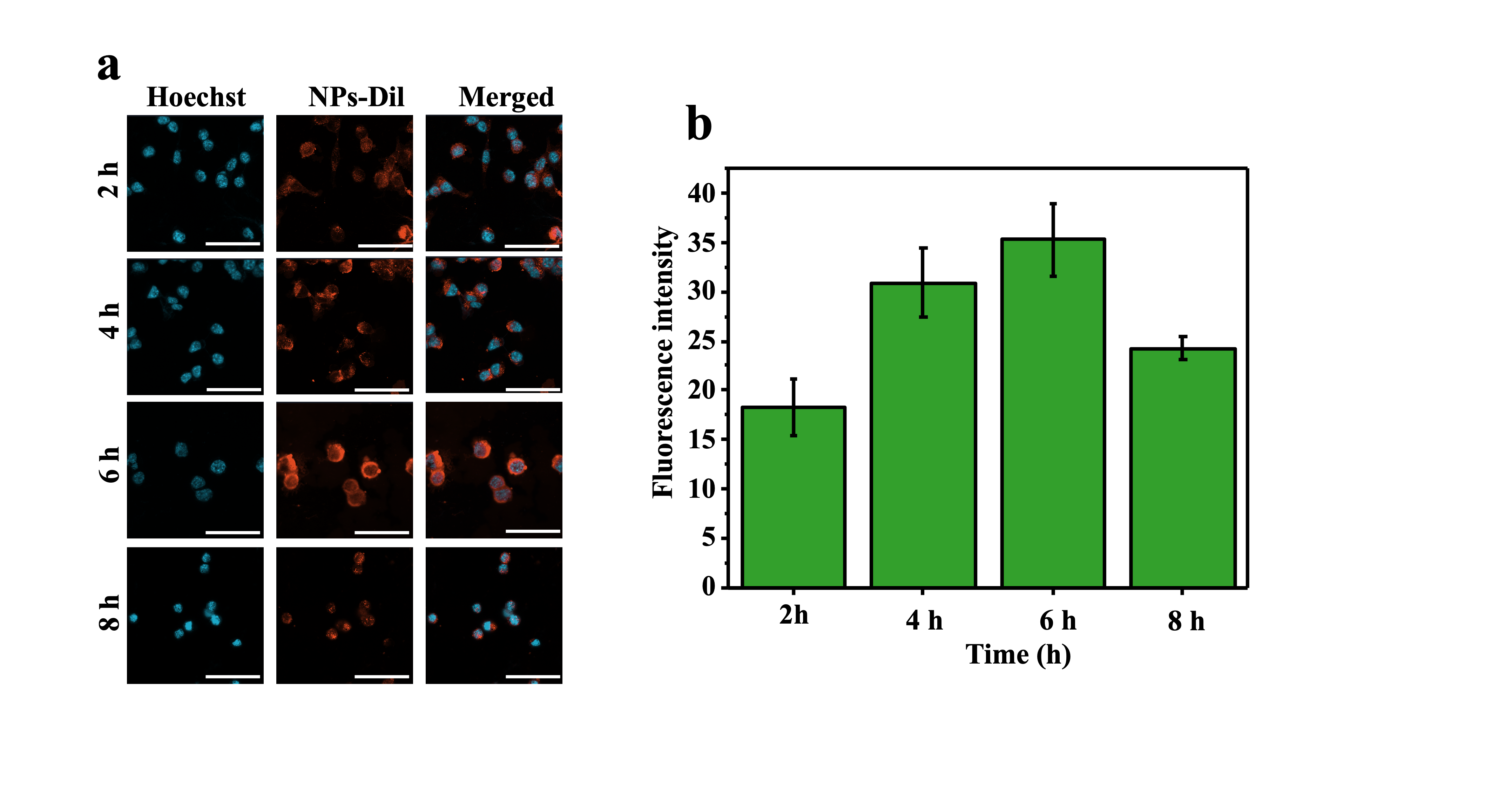


**Fig. S4.** (a) Fluorescence images of CT26 cells treated with DiI labelled CuSe@albumin NPs over different time intervals. Cell nuclei were identified with Hoechst 33342. CuSe@albumin = 75 μg/ml and (b) quantified data of the fluorescence intensity. Scale bar = 50 μm.

**
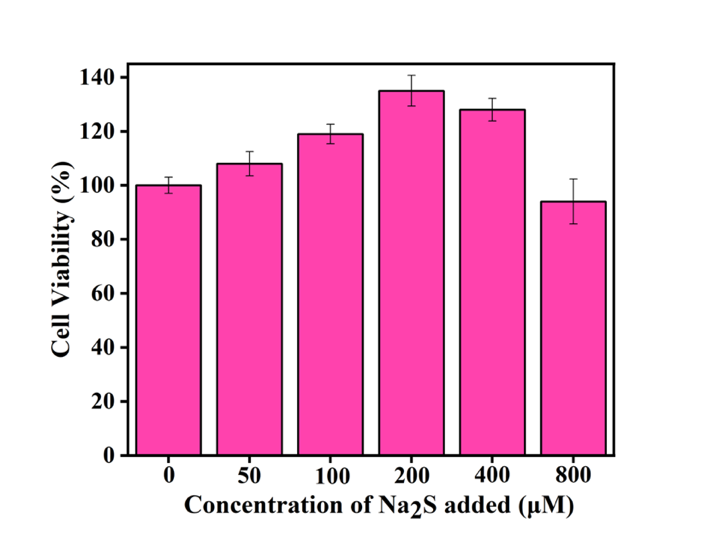
**

**Fig. S5.** Cell viability of CT26 cells treated with H_2_S (Na_2_S) of varying concentrations.


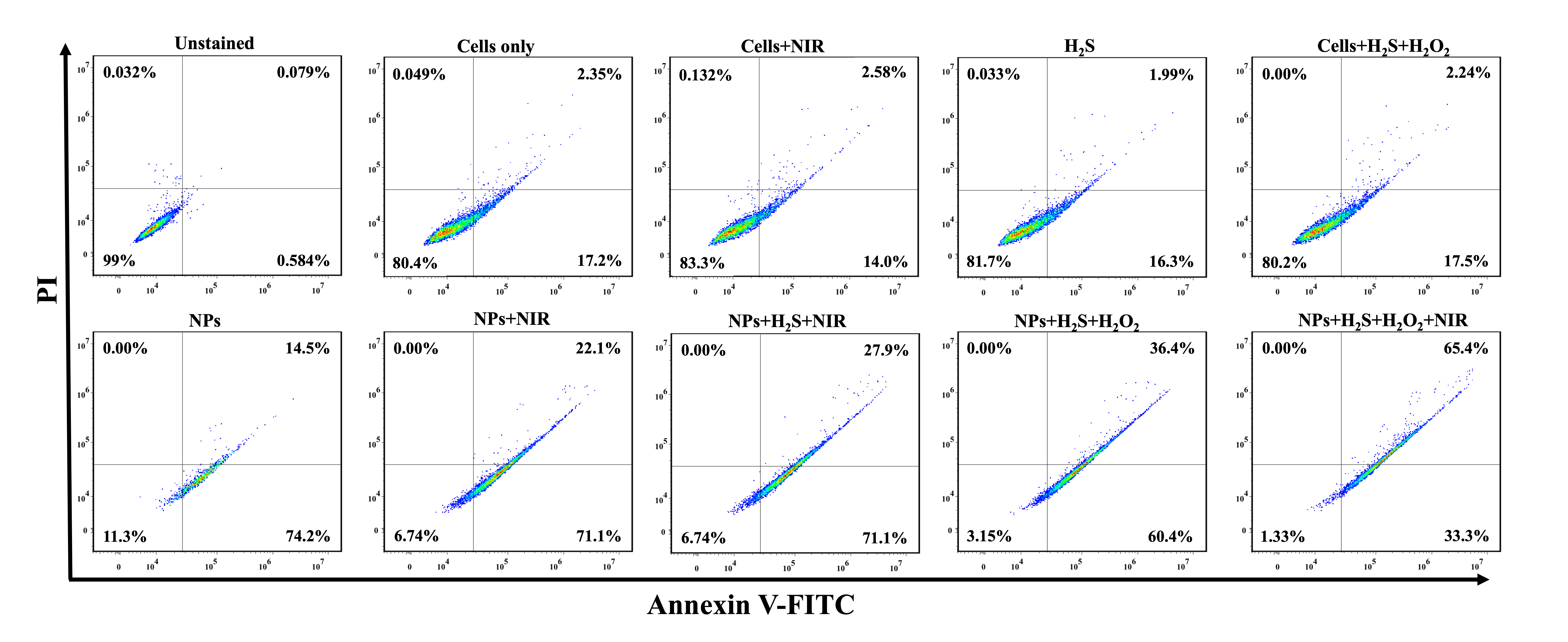


**Fig. S6.** Apoptosis detection of CT26 cells by flow cytometry with Annexin V-FITC and PI staining after different treatments.


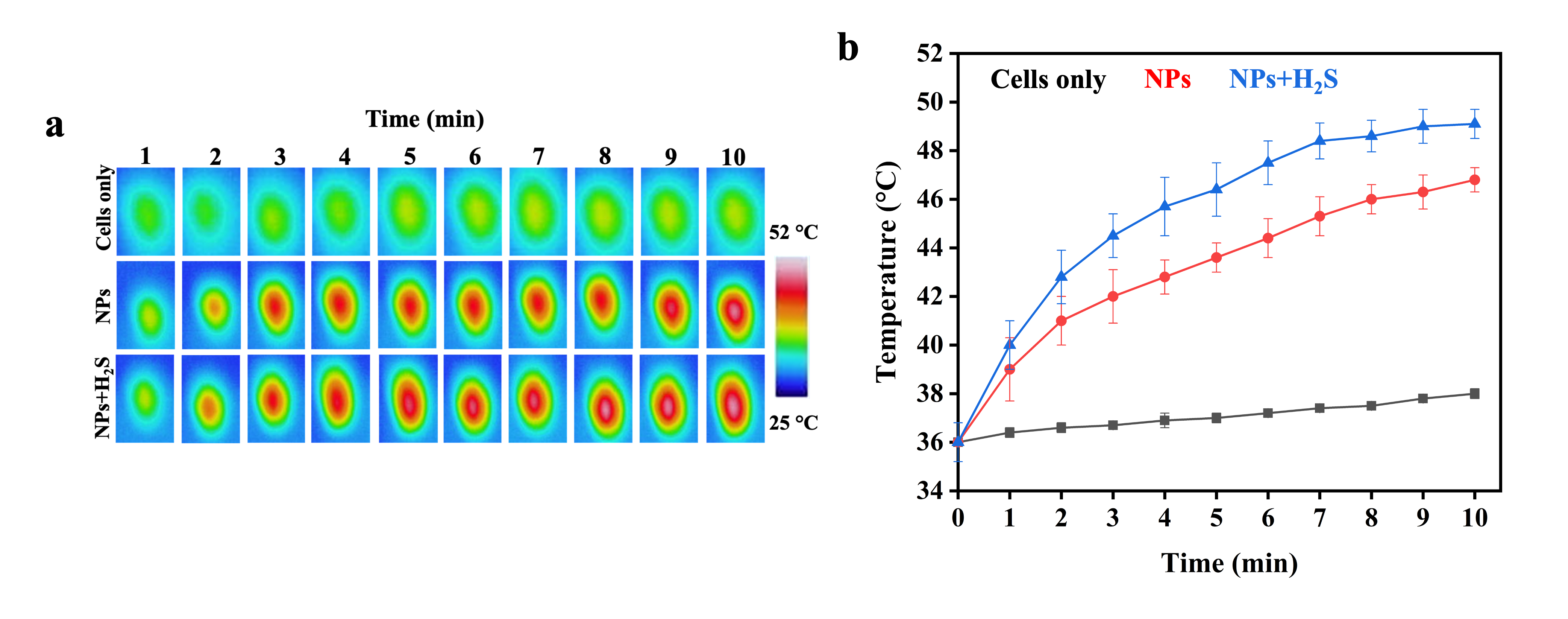


**Fig. S7.** (a) Thermal images of CT26 cells treated separately with CuSe NPs and NPs+H_2_S, followed by NIR irradiation for 10 min. (b) The temperature profiles of CT 26 cancer cells attained by the IR thermal imaging over 10 min.


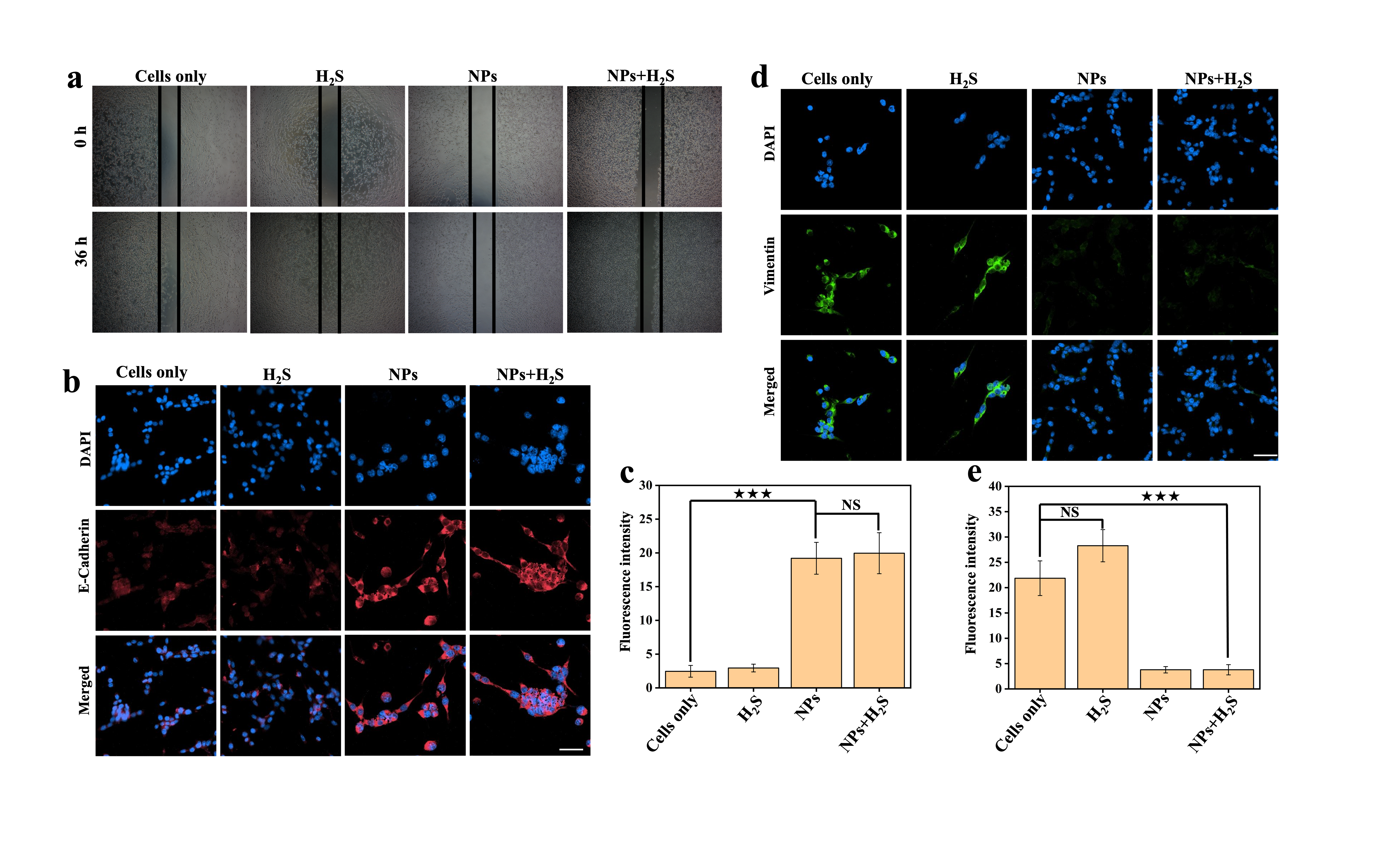


**Fig. S8.** (a) Cell migration of CT26 cancer cells after receiving different treatments as evaluated by wound healing assay. Immunofluorescence images and quantified data of the fluorescence intensity of (b, c) E-cadherin (red) and (d, e) vimentin (green) in CT26 cells after different treatments. Scale bars 50 μm; *P < 0.05, **P < 0.01, ***P < 0.001.


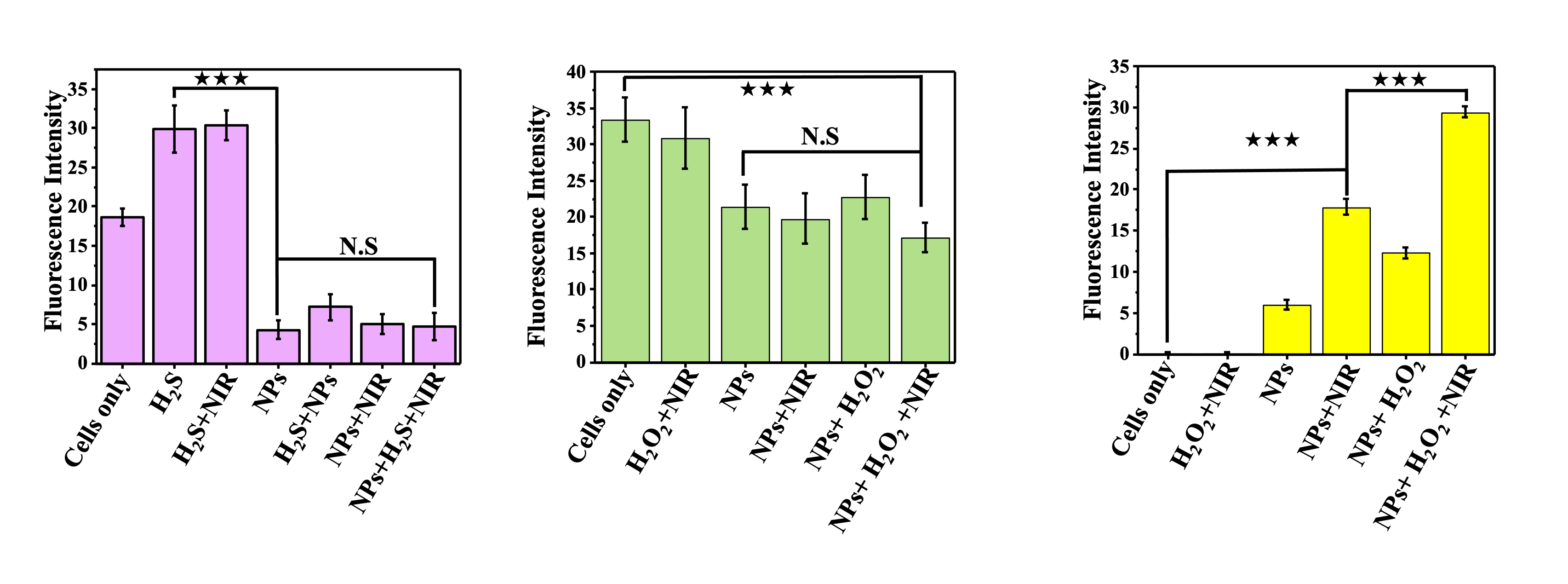


**Fig. S9.** Quantitative data of (a) H_2_S (b) GSH and (c) ROS levels in CT26 cells after different treatments attained from the fluorescence images in Fig. 4d, f, and h, respectively. *P < 0.05, **P < 0.01, ***P < 0.001.


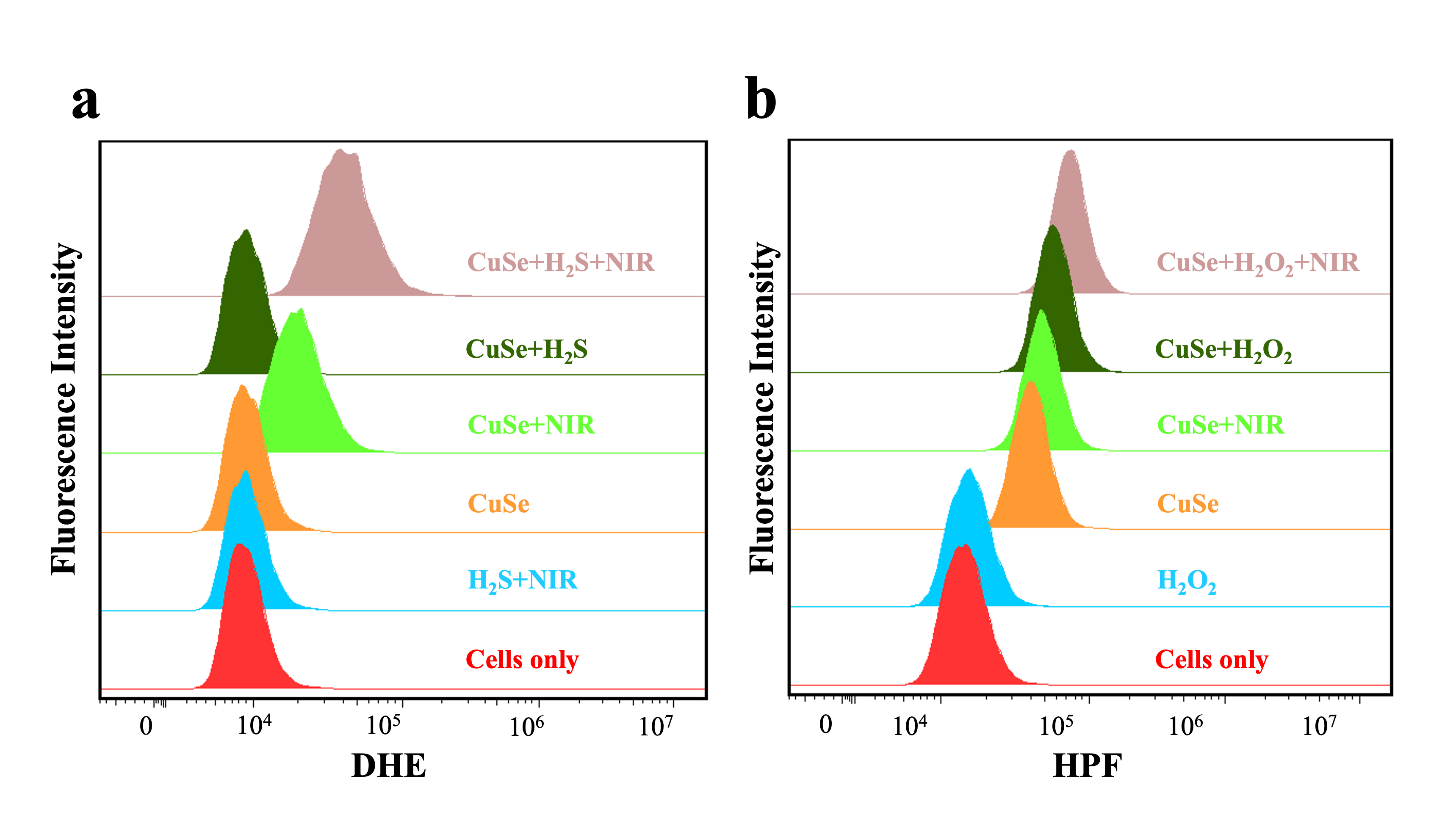


**Fig. S10.** Flow cytometric detection of (a) O_2_^•-^ radicals using DHE and (b) •OH radicals with HPF in CT 26 cells after different treatments.

**
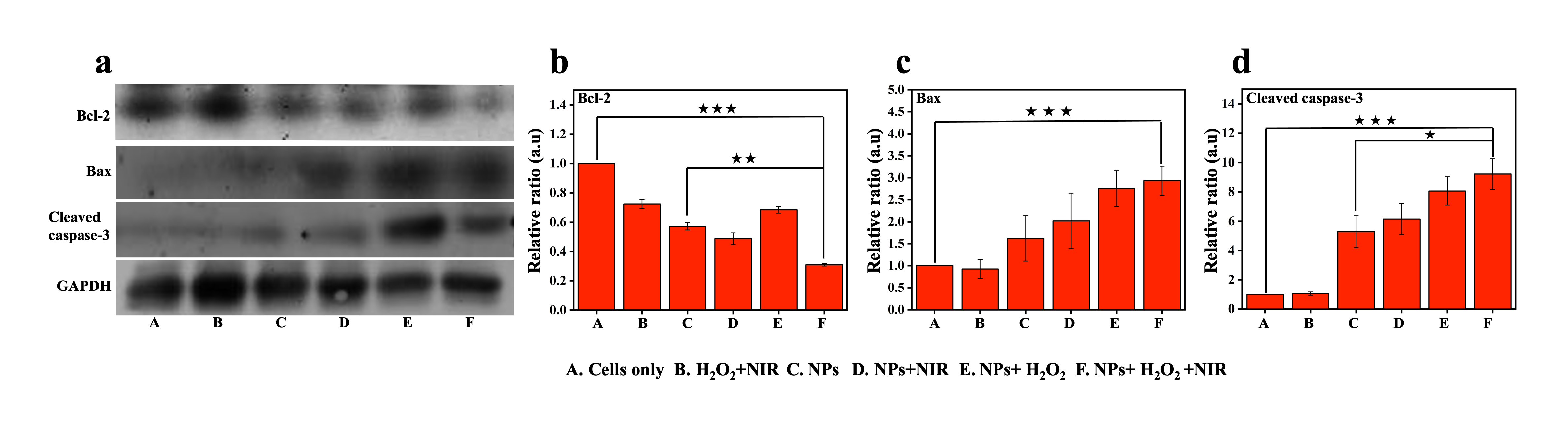
**

**Fig. S11.** a) Western blot evaluation of apoptosis-related proteins (Bcl-2, Bax, and cleaved caspase-3) in CT26 cells after different treatments. Quantitative data for b) Bcl-2, c) Bax, and d) cleaved caspase-3 normalized to β-actin. *P < 0.05, **P < 0.01, ***P < 0.001.


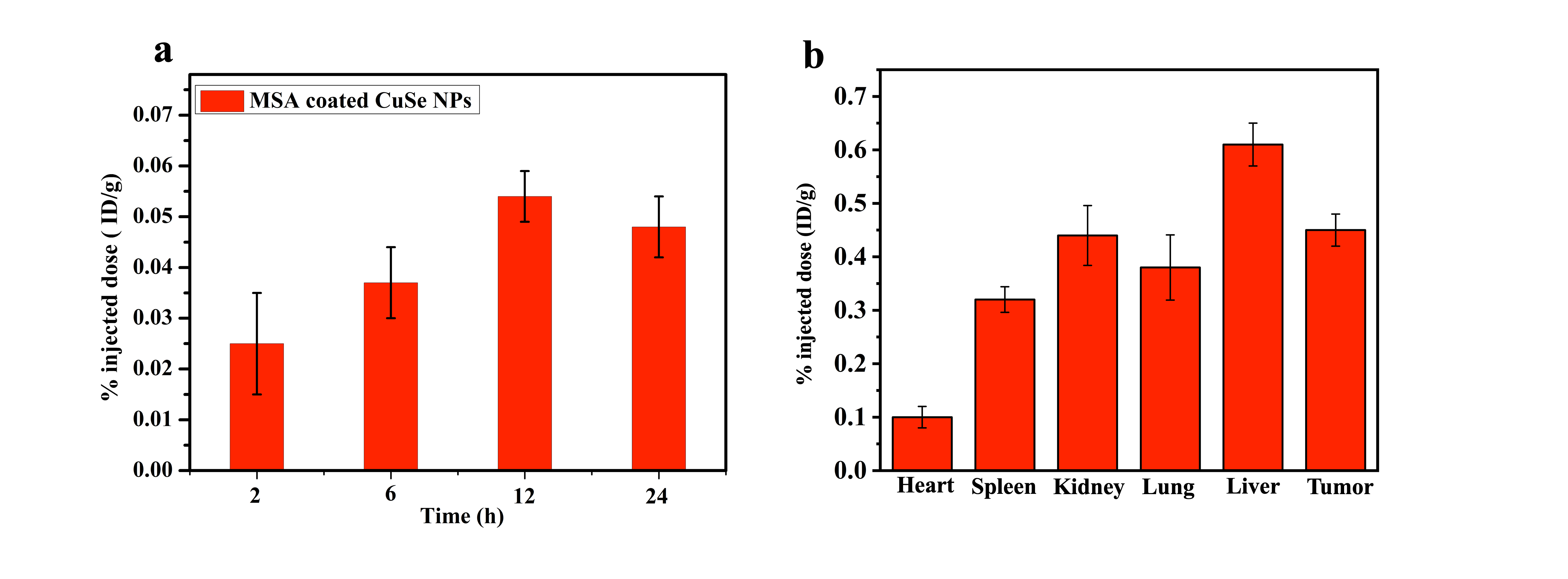


**Fig. S12. (**a) Time-evolved accumulation of NPs in tumor. (b) Biodistribution of NPs in major organs and tumor at 12 h post IV injection. The measurement was performed by ICP-MS.


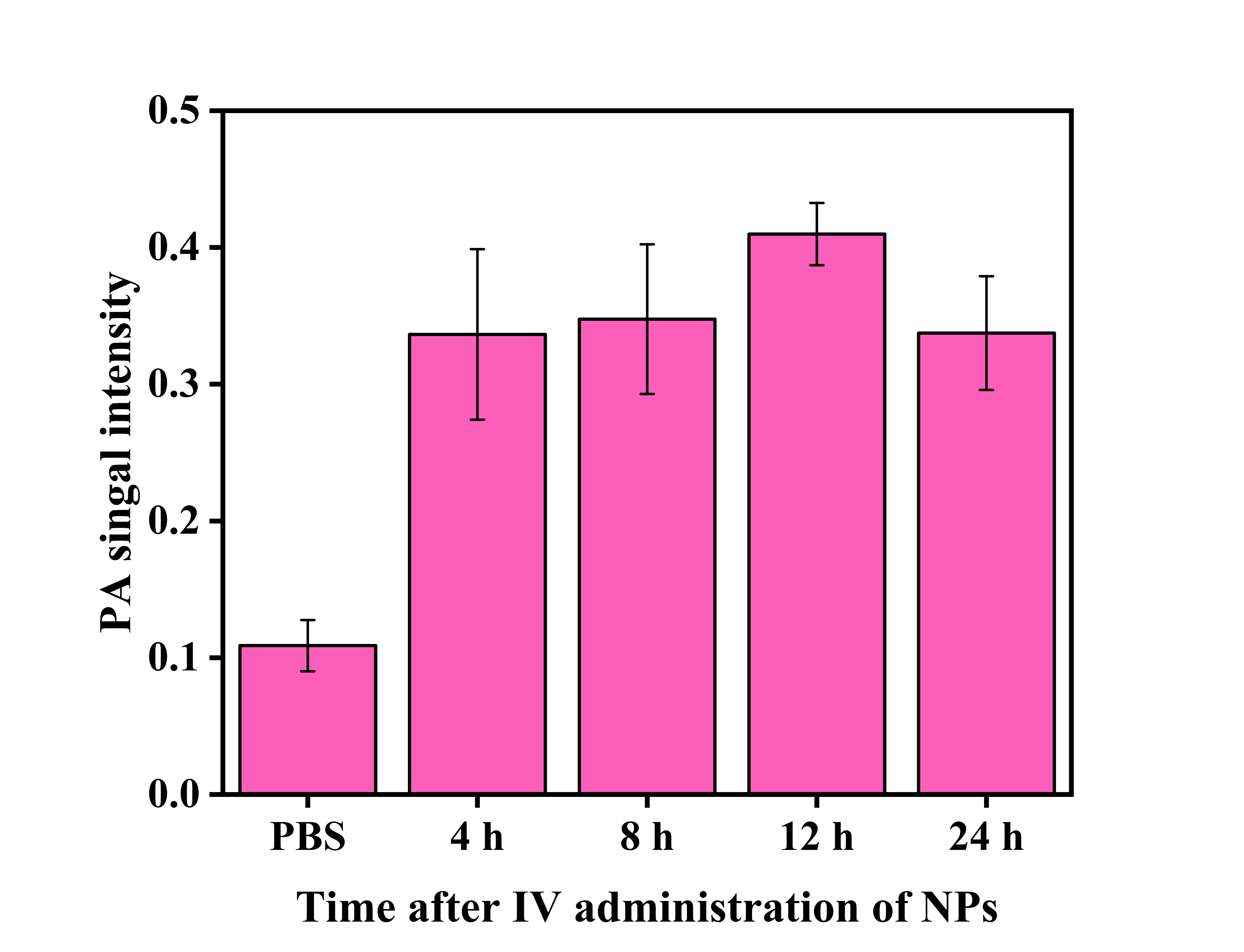


**Fig. S13.** Quantitative data of the PA signal intensity obtained from PA imaging (Fig. 5b) of tumor regions at different time points after IV administration of NPs.


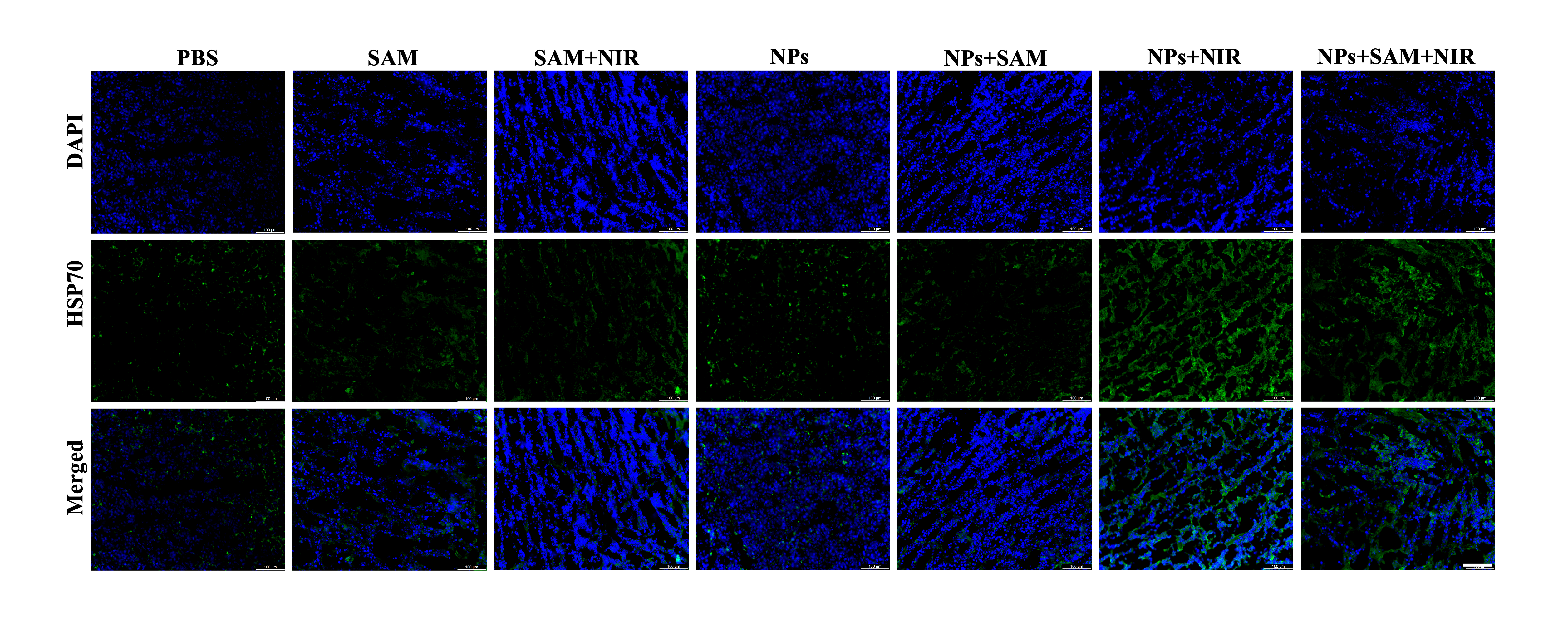


**Fig. S14.** Immunofluorescence staining of HSP70 in tumor tissues excised on day 14 after different treatments. Scale bar 100 µm

**
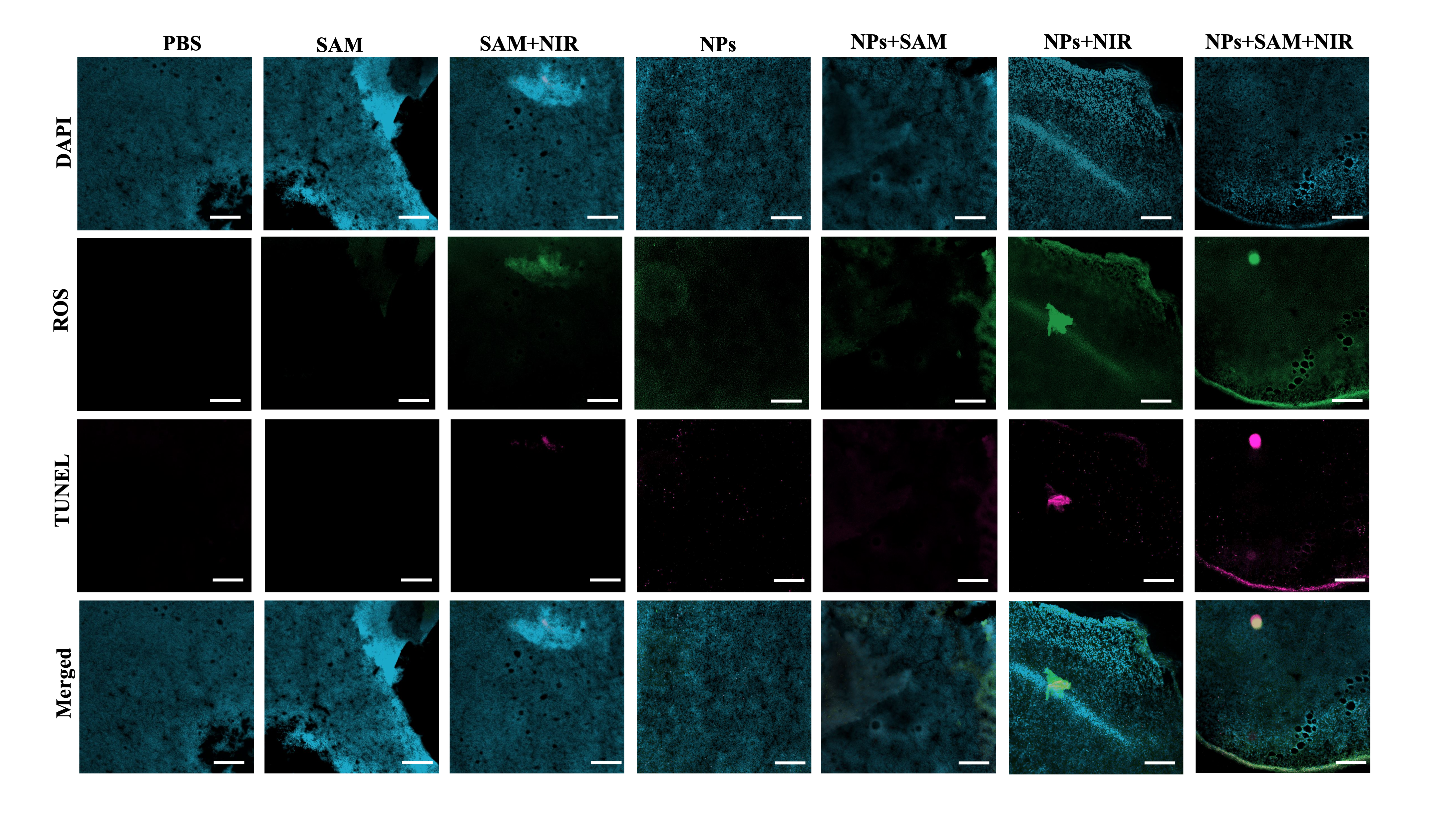
**

**Fig. S15.** DCFH-DA (ROS) and TUNEL (DNA damage) staining of tumor tissues after different treatments. Scale bar 100 µm

**Fig. S16. (**a) H_2_S staining with WSP-1 of tumor sections on day 14 after the treatments. (b) Immunofluorescence images of tumor tissue stained with endothelial CD31 marker in red. Scale bar 100 μm. DAPI in blue was used to identify cell nuclei.


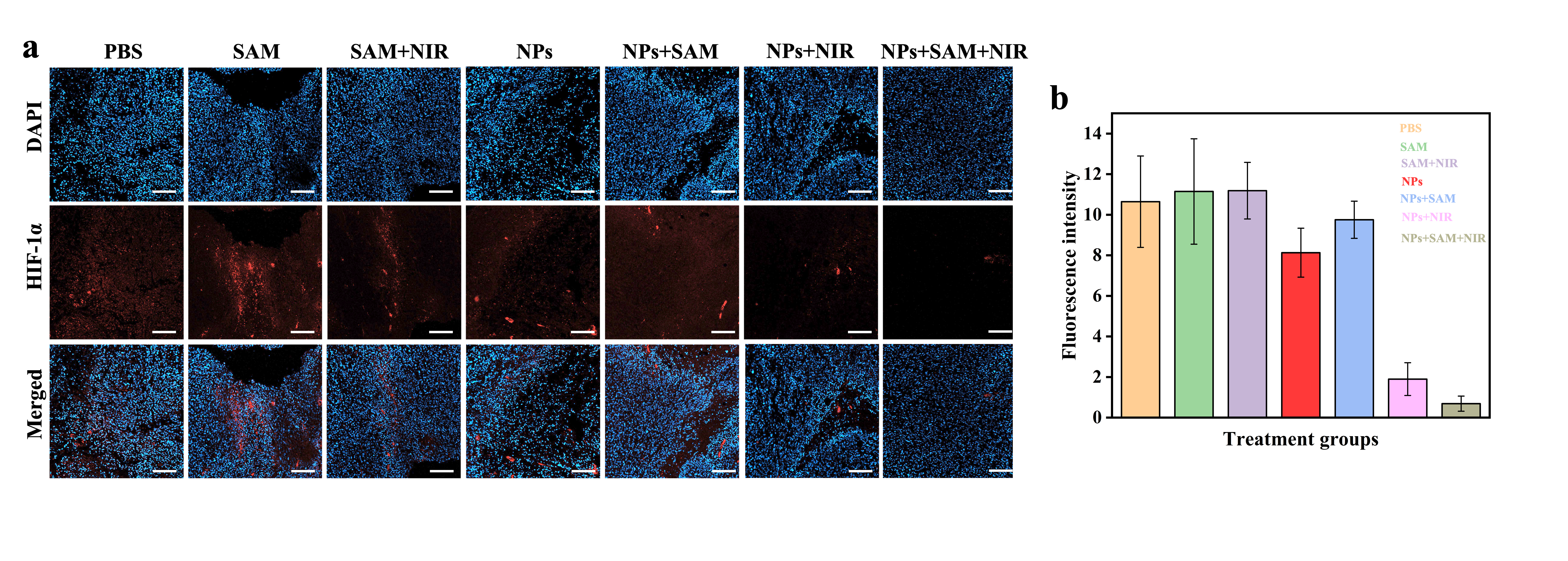


**Fig. S17.** (a) Fluorescence images attained by immunofluorescence staining and (b) quantitative analysis of HIF-1α expression in tumor sections after different treatments. Scale bar 100 µm


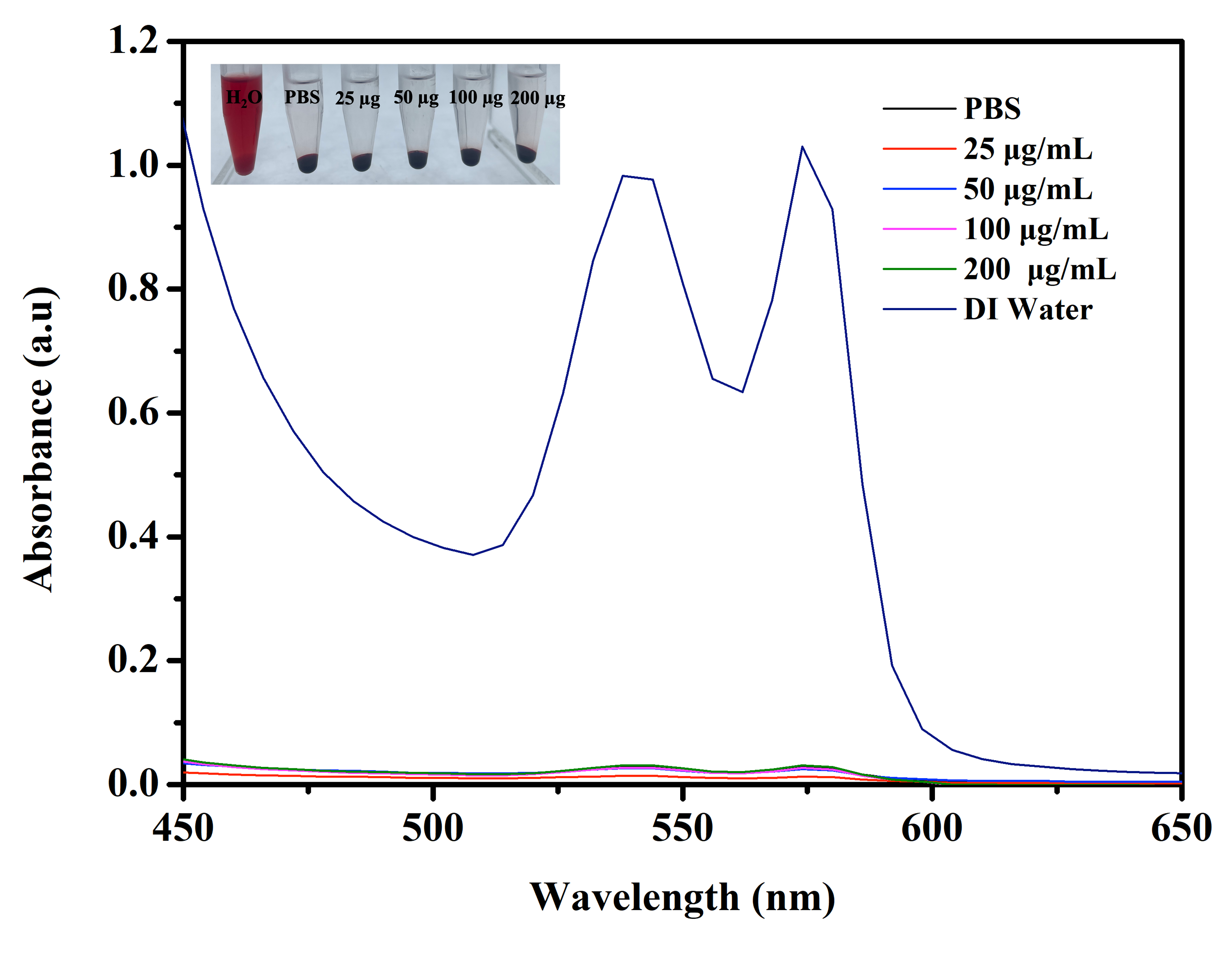


**Fig. S18.** Hemolytic activity measurements of CuSe NPs with varying concentrations. The inset photos are the centrifuged RBCs in blood after the treatments.

**
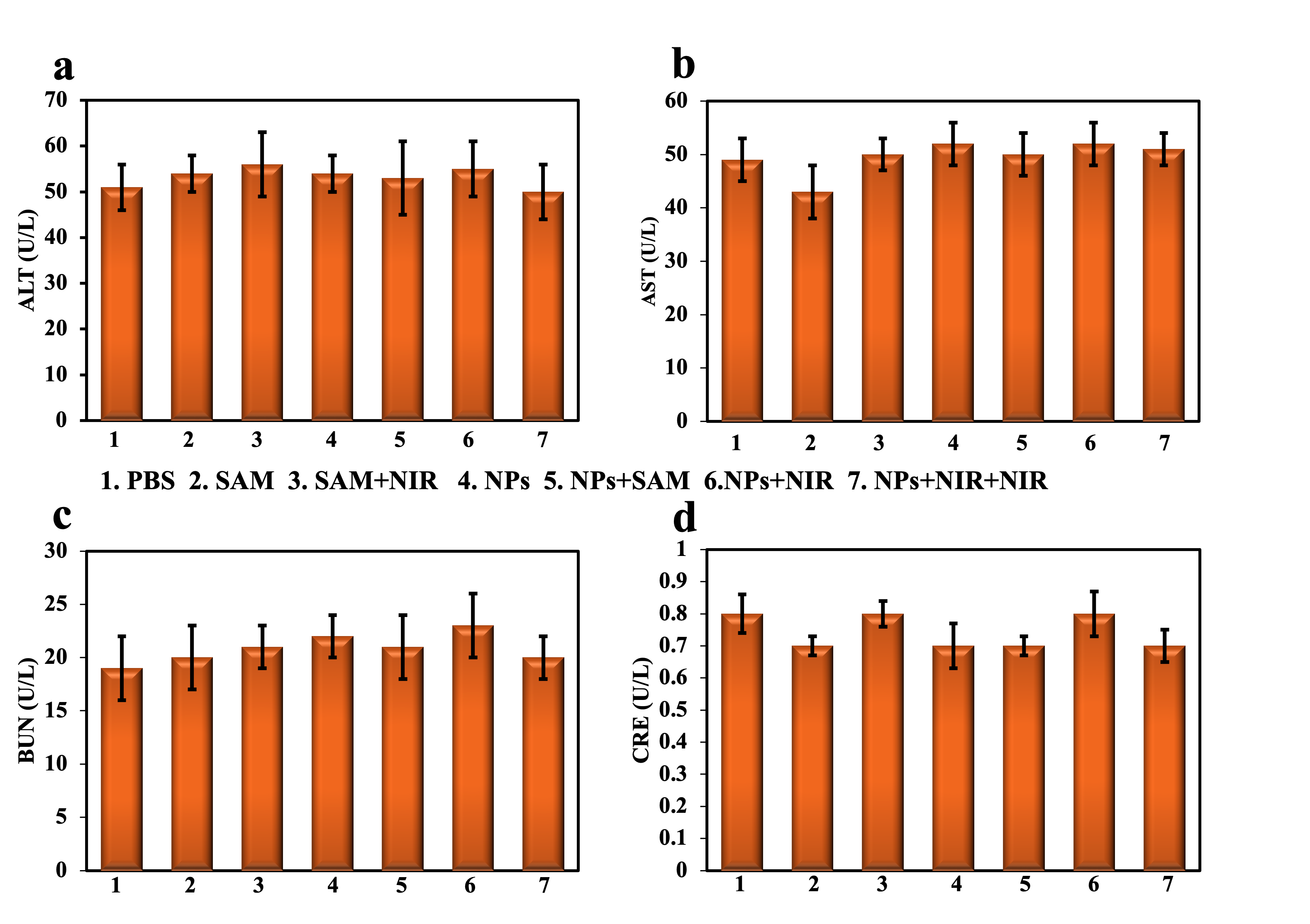
**

**Fig. S19.** Blood biochemistry assay from tumor-bearing mice after IV administration of MSA-coated CuSe NPs (two doses), including (a) ALT, (b) AST, (c) BUN, and (d) CRE. n=3.
